# Association of surgeons’ gender with elective surgical lists in the State of Florida is explained by differences in mean operative caseloads

**DOI:** 10.1371/journal.pone.0283033

**Published:** 2023-03-15

**Authors:** Franklin Dexter, Richard H. Epstein, Brenda G. Fahy

**Affiliations:** 1 Division of Management Consulting, Department of Anesthesia, University of Iowa, Iowa City, Iowa, United States of America; 2 Department of Anesthesiology, Perioperative Medicine & Pain Management, Miller School of Medicine, University of Miami, Miami, Florida; 3 Department of Anesthesiology, University of Florida, Gainesville, Florida; Stanford University School of Medicine, UNITED STATES

## Abstract

**Background:**

A recent publication reported that at three hospitals within one academic health system, female surgeons received less surgical block time than male surgeons, suggesting potential gender-based bias in operating room scheduling. We examined this observation’s generalizability.

**Methods:**

Our cross-sectional retrospective cohort study of State of Florida administrative data included all 4,176,551 ambulatory procedural encounters and inpatient elective surgical cases performed January 2017 through December 2019 by 8875 surgeons (1830 female) at all 609 non-federal hospitals and ambulatory surgery centers. There were 1,509,190 lists of cases (i.e., combinations of the same surgeon, facility, and date). Logistic regression adjusted for covariables of decile of surgeon’s quarterly cases, surgeon’s specialty, quarter, and facility.

**Results:**

Selecting randomly a male and a female surgeons’ quarter, for 66% of selections, the male surgeon performed more cases (P < .0001). Without adjustment for quarterly caseloads, lists comprised one case for 44.2% of male and 54.6% of female surgeons (difference 10.4%, P < .0001). A similar result held for lists with one or two cases (difference 9.1%, P < .0001). However, incorporating quarterly operative caseloads, the direction of the observed difference between male and female surgeons was reversed both for case lists with one (-2.1%, P = .03) or one or two cases (-1.8%, P = .05).

**Conclusions:**

Our results confirm the aforementioned single university health system results but show that the differences between male and female surgeons in their lists were not due to systematic bias in operating room scheduling (e.g., completing three brief elective cases in a week on three different workdays) but in their total case numbers. The finding that surgeons performing lists comprising a single case were more often female than male provides a previously unrecognized reason why operating room managers should help facilitate the workload of surgeons performing only one case on operative (anesthesia) workdays.

## Introduction

Managerial epidemiological studies of anesthesia and surgery in the USA have consistently found low productivity of most hospital surgical suites and ambulatory surgery centers (i.e., there is substantial unused time) [1–6]. Many surgical facilities nationwide have far fewer than 8 hours of cases per anesthetizing location per workday [1–6], especially at anesthetizing locations that are not operating rooms [5, 6]. The many surgeons who perform one case on days they operate and those who perform, on average, two or fewer cases per week, have reduced productivity that results from waiting on the day of surgery for completion of preceding surgical cases that take more time than originally estimated [7]. There are many such surgeons, given that previous studies have shown that most surgeons in both Florida and Iowa perform only one or two cases on the days they operate [8–10]. This finding is unaltered by whether the surgeon principally cares for adult or pediatric patients [8–10]. Frequently, operating room managers do not plan convenient access to operating room time for low-caseload surgeons. This deficiency has been shown in multiple studies with different populations to result principally from lack of scientific knowledge of mathematically optimal surgical scheduling strategies among the decision-makers [11–16]. Typically, managers reserve too much time for surgeons with high daily caseloads and too little time, collectively, for surgeons with low daily caseloads [11, 17]. Courses in the relevant management science significantly increase trust in the relevant mathematical techniques and improve managerial skill in their application [14, 15, 18–20].

An earlier survey study by Yesantharao et al. suggested a novel additional mechanism for why surgeons performing single cases on operative days may have insufficient access to operating room time [21]. They found, at three affiliated teaching hospitals, that female surgeons were less likely than male surgeons to have adequate operating room block time compared to their reported hourly workloads (multivariable-adjusted P = .004) [21]. In other words, implicit or explicit gender-based bias of the operating room managers could be a contributing factor.

We performed a managerial epidemiology study to examine the generalizability of this observation of possible gender bias concerning surgeon access to operating room time across all ambulatory surgery centers and (non-federal) hospitals in Florida [22]. We compared lists of cases (i.e., combinations of a surgeon, date, and facility) between female and male surgeons. We tested whether female surgeons more often than male surgeons performed only one case on days where they did at least one elective surgical case (i.e., had lists of one case). Following Yesantharo [21], our hypothesis #1 is that, overall, female surgeons would have a greater fraction of their lists with one case. This comparison of lists with one case is measurable because many surgeons perform just one elective case on a regular (non-weekend, non-holiday) workday: 47.2% of 175,751 lists among hospitals in Iowa [8], 47.4% of 1,114,860 lists among hospitals and ambulatory surgery centers in Florida [9], and 47.9% of 27,557 lists among pediatric hospitals [10]. This comparison is relevant because observations of multiple populations have found no surgical specialty to have average case durations sufficiently long that single cases would usually fill an operating room for the workday [23–26]. Hypothesis #1 is important because although these low-caseload surgeons account for most surgical growth from year to year [10, 25, 26], their individual percentage utilization of operating room time, either adjusted (including turnover times) or raw (without turnover times) [27], cannot be measured accurately [23, 28]. Such limitations associated with measuring workload of low-caseload surgeons is an example of the type of knowledge necessary for teams responsible for operating room time to make the best possible decisions [11, 13–15].

If there were differences in the percentages of lists with one case between female and male surgeons, there would be different work experiences of female and male surgeons in surgical suites [7, 21, 29]. Understanding the causes of gender-related differences in case scheduling would give insight to operating room managers on how to address potential gender-based concerns related to inequality of access to operating room time. For example, the raw differences between genders might be an artifact from including surgeons’ specialty as a confounder, but not considering surgeons’ quarterly operative caseloads [22]. Our hypothesis #2 was that gender differences would remain despite incorporating confounders, most specifically, quarterly caseloads. If hypothesis #2 were rejected, an implication would be that single-case lists are not related to gender bias, but rather to other factors, such as smaller quarterly caseloads.

## Materials and methods

The Institutional Review Board of the University of Florida (IRB202002442) approved this research as exempt from patient consent. The Institutional Review Boards of the University of Iowa (October 20, 2021) and the University of Miami (October 21, 2021) determined that the current analyses of de-identified data do not meet the regulatory definition of human subjects research.

### Cross-sectional retrospective cohort study using State of Florida data

We used publicly available data from the Agency for Health Care Administration (AHCA) for patients receiving care at non-federal hospitals and ambulatory surgery centers in Florida from January 1, 2017, through December 31, 2019 [22]. Following approval by AHCA, these data were supplemented with the date of each encounter (for ambulatory patients) and the date of each hospital admission (for inpatients). Dates were necessary to obtain our primary endpoint, the percentage of daily lists of cases (i.e., combinations of a surgeon, facility, and date of surgery) per quarter that included one case. Such discharge abstract data do not include case durations or surgical times, but even if they did, percentage utilizations could not be estimated accurately for these surgeons [23, 28]. Data use agreements were executed between the University of Florida and AHCA, and between the University of Florida and the University of Miami. AHCA disclaims responsibility for the results and conclusions of the study. Sharing of these data is precluded by those data use agreements; readers interested in analyzing the raw data will need to apply for their use with the AHCA as done by the authors.

Table 1 shows the exclusion criteria among the 10,589,761 outpatient encounters and inpatient elective surgical admissions in Florida included in this study [22]. The resulting 4,176,551 cases were performed during the 12 included quarters by 8875 surgeons, with female (21%) or male gender determinable for all surgeons from the National Provider Identifier database (Table 2) [30]. Throughout our paper, we use the terms “male” and “female” because non-binary options for gender are not provided in the study database.

**Table 1:** Flow diagram of elective case ^a^ exclusions from the 287 hospitals and 440 ambulatory surgery centers in Florida, 2017–2019.

Total Cases Remaining from Initial 10,569,761	Outpatient Cases Excluded^b^ from Initial 9,444,625	Inpatient Cases Excluded^b^ from Initial 1,125,136	Both Types of Cases Excluded^b^^,^^c^	Exclusion Criteria
4,800,667	5,394,312	374,782		Outpatient: All CPT codes for the visit have 0 intraoperative wRVU or 0 ASA base units Inpatient: Primary ICD-10-PCS associated with the admission was a minor therapeutic or minor diagnostic procedure
4,774,412	17,367	8888		Case performed on a weekend or federal holiday
4,610,755	158,489	5168		Missing or invalid NPI, or the NPI was not for a physician, oral surgeon/dentist, or podiatrist
4,242,899			367,856	Non-surgical specialty was associated with the NPI of the performing provider
4,233,337			9562	Case’s date was earlier than the date of the first quarter where the surgeon did at least three cases or later than the last quarter when surgeon did at least three cases^d^
4,176,551			56,786	Surgeon did not have at least three quarters with at least one case
4,176,551	5,570,168	388,838	434,204	All exclusion criteria, resulting in cases of 8875 surgeons performed at 609 facilities

Abbreviations: ASA, American Society of Anesthesiologists; CPT Current Procedural Terminology; ICD-10-PCS International Classification of Diseases, 10^th^ Revision, Procedure Coding System; NPI, national provider identifier; wRVU, work relative value units.

^a^ Elective cases were (i) all ambulatory encounters with total wRVU>0 and ASA base units >0, plus (ii) hospital admissions where the principal procedure was performed on the date of admission, the priority of the admission was elective, and there were no emergency department charges for the admission

^b^ Exclusions in each row were applied sequentially. Thus, the numbers listed other than in the first cell in the column are not the number of cases meeting the exclusion criteria, but rather the number excluded after all prior exclusions were applied.

^c^ Exclusions in this column were applied to the combined inpatient and outpatient cases because surgeons typically operate on both categories of patients. These criteria were made based on criteria related to the surgeon.

^d^ We aimed to study active surgeons. The quarters studied were calendar quarters reflecting the administrative data. Thus, we excluded the surgeon who performed only one case during one quarter. For example, if the surgeon had started a new job, low caseload would be an artifact of their starting their surgical practices and would have biased the results.

**Table 2:** Gender distribution of surgeons, surgeon quarters, cases, and lists.

Criteria	N	Female % ^a^
Surgeons	8875	21%
Surgeon quarters, there being 12 quarters studied^b^	94,005	20%
Cases	4,176,551	12%
Lists of cases (i.e., combination of surgeon, facility, and date)	1,509,190	14%
Deciles among surgeon lists, the deciles of surgeon quarters and cases provided in the supplemental content		
<5 cases per quarter, median 2	22,219	31%
5 to 8 cases per quarter, median 6	54,449	34%
9 to 13 cases per quarter, median 11	91,024	31%
14 to 18 cases per quarter, median 16	100,886	24%
19 to 25 cases per quarter, median 22	138,368	18%
26 to 34 cases per quarter, median 30	162,647	13%
35 to 47 cases per quarter, median 41	197,474	12%
48 to 66 cases per quarter, median 56	224,704	11%
67 to 101 cases per quarter, median 82	254,787	9%
>101 cases per quarter, median 184	262,632	8%
Primary surgical specialty by surgeon lists, with distribution by surgeon quarters, surgeons, and cases provided in the supplemental content		
Orthopedic surgery	340,921	4%
General surgery	258,436	14%
Obstetrics/ gynecology	192,176	41%
Ophthalmology	164,787	18%
Urology	108,242	4%
Otolaryngology	88,512	14%
Plastic surgery	74,672	13%
Neurosurgery	71,938	4%
Podiatric surgery	55,841	14%
Cardiothoracic surgery	44,202	2%
Vascular surgery	34,605	8%
Colorectal surgery	5,459	17%
Gynecological oncology	17,048	37%
Surgical oncology	14,600	25%
Oral maxillofacial surgery and intraoperative dental care	7447	6%
Gastroenterology	4413	13%

^a^ Each surgeon’s gender was identified as listed in National Provider Identifier database. The percentages are reported with too few digits to reconstruct the counts without error. The raw counts are included in the supplemental content, listed in the sequence of this table.

^b^ “Surgeon quarters” refer to combinations of surgeons and quarters. With 8875 surgeons and 12 quarters there could have been a maximum 106,500 combinations, where 106,500 = 8875 × 12, but 94,005 were observed (i.e., there were some quarters when they did not operate). The supplemental content includes the distribution between genders for each quarter among surgeon quarters, cases, and lists of cases.

### Statistical analyses

Full statistical output, including commands and comments, is provided in the supplemental content. These are listed in the same sequence of the Methods and Results for readers interested in more detail or who want to replicate our work with other state or provincial datasets. Stata v17.0 was used for all analyses (StataCorp, College Station, Texas).

Univariate analyses were performed to compare surgeons based on gender. Exact binomial confidence intervals for the area under the receiver operating characteristic curves (c-statistic) of each surgeon’s quarterly operative caseload to predict gender [31, 32] were calculated. The 99% confidence intervals for the proportion of lists that comprised one case were calculated using the delta method.

Hypothesis #1 assessed the unadjusted association between surgeon gender and whether the list contained one case. Logistic regression was used, P < .01 treated as significant.

Hypothesis #2 assessed adjusted analyses. Logistic regression was used to control for deciles of cases per quarter, specialty, quarter, and interactions between gender and deciles (Tables 2 and 3). We applied clustering by facility when calculating the standard errors. The supplemental content includes rationales and details related to the calculations, and the associated regression coefficients from the logistic regressions. P < .01 was treated as significant.

**Table 3:** Percentages of lists of cases that included one case or one or two cases, with 99% confidence intervals ^a,b^.

Criteria	One case	One or two cases
Overall	46% (45% to 46%)	67% (67% to 67%)
Male surgeons	44% (44% to 44%)	66% (65% to 66%)
Female surgeons	55% (54% to 55%)	75% (74% to 76%)
Surgeons’ numbers of cases during the quarter within each decile, with corresponding estimates by combination of surgeon’s gender and cases during the quarter given in the supplemental content		
<5 cases per quarter, median 2	94% 93% to 94%)	99% (99% to 99%)
5 to 8 cases per quarter, median 6	86% (85% to 86%)	98% (98% to 98%)
9 to 13 cases per quarter, median 11	79% (78% to 79%)	96% (95% to 96%)
14 to 18 cases per quarter, median 16	73% (72% to 73%)	93% (93% to 93%)
19 to 25 cases per quarter, median 22	66% (66% to 67%)	89% (89% to 90%)
26 to 34 cases per quarter, median 30	58% (58% to 59%)	85% (84% to 85%)
35 to 47 cases per quarter, median 41	49% (48% to 49%)	77% (77% to 78%)
48 to 66 cases per quarter, median 56	38% (38% to 39%)	66% (65% to 66%)
67 to 101 cases per quarter, median 82	27% (26% to 27%)	50% (49% to 50%)
>101 cases per quarter, median 184	15% (14% to 15%)	26% (25% to 26%)
Surgeon’s primary specialty, with corresponding estimates by combination of surgeon gender and specialty given in the supplemental content		
Orthopedic surgery	32% (32% to 33%)	53% (53% to 54%)
General surgery	43% (43% to 44%)	68% (68% to 69%)
Obstetrics/ gynecology	72% (71% to 72%)	91% (91% to 91%)
Ophthalmology	19% (19% to 20%)	30% (30% to 31%)
Urology	54% (54% to 55%)	78% (78% to 79%)
Otolaryngology	42% (42% to 43%)	65% (64% to 66%)
Plastic surgery	55% (54% to 56%)	78% (77% to 79%)
Neurosurgery	49% (48% to 51%)	77% (76% to 78%)
Podiatric surgery	63% (62% to 64%)	85% (84% to 86%)
Cardiothoracic surgery	74% (72% to 75%)	94% (94% to 95%)
Vascular surgery	59% (58% to 60%)	84% (82% to 85%)
Colorectal surgery	48% (47% to 50%)	74% (72% to 75%)
Gynecological oncology	36% (34% to 38%)	61% (58% to 64%)
Surgical oncology	47% (44% to 49%)	74% (71% to 76%)
Oral maxillofacial surgery and intraoperative dental care	71% (68% to 74%)	91% (89% to 93%)
Gastroenterology	70% (65% to 74%)	87% (84% to 91%)

^a^ The percentages are reported to the nearest 1%. The estimates are reported with many more digits in the supplemental content. The supplemental content and this table are presented in the same sequence.

^b^ The supplemental content includes the estimated ratios, one case or one or two cases, for each of the 12 quarters.

Both hypotheses were formulated in terms of differences of proportions. (Although odds ratios were calculated, they grossly overstate relative risks because the prevalence of surgeons having lists of one case, or one or two cases, was approximately 50%, not small [e.g., 5%] or large [e.g., 95%] (Table 3). Therefore, we report the odds ratios only in supplemental content.) Contrasts of predictive probabilities between genders were calculated using the STATA *margins* command. Numerical variables were handled in the regression analyses by using their average estimates over all observations. Confidence intervals were calculated using the delta method to apply the regression model’s variance estimates. These contrasts are reported in the paper because they combine the coefficients of gender alone and the interactions in the nonlinear (logistic) regression.

Several sensitivity analyses were performed. First, individual independent variables (e.g., cases per quarter) were removed from the models to understand what adjustments were influencing results. Second, the dependent variable was changed from one case per list to one or two cases per list (Table 3). Third, we examined sensitivity of confidence interval width (i.e., statistical power) to the numbers of lists (i.e., hypothetical benefit of adding years of data) versus the numbers of facilities (i.e., would need to add more US state[s]).

Regarding study size, in addition to the preceding sensitivity analysis, we also evaluated our decision to proceed (i.e., test our hypothesis #1) after reading the report by Yesantharao from three hospitals [21]. To judge minimum differences that are managerially important, the University of Iowa Department of Anesthesia routinely holds meetings addressing requirements to provide anesthesia services for procedural based physicians who will do 1 or 2 cases in a procedural suite every two weeks (e.g., pain medicine physicians) [33]. Because such proceduralists generally can fill an operating room for half-day, managers regularly treat issues related to 1 out of 20 lists as appropriately calling for their attention [33], where 20 = (10 days in 2 weeks) × (2 of these brief lists per workday per anesthesia practitioner). Being conservative, statistically, if the 99% confidence interval (P < .01) for the unadjusted absolute difference between female and male surgeons excluded 5% (i.e., 1 out of 20 lists), then the study size would be sufficiently large to detect a managerially important difference at the University of Iowa. Such considerations underestimate value because there also are organizational and societal consequences for policies being biased (e.g., based on gender).

## Results

We analyzed 1,509,190 lists of cases performed by 8875 surgeons (Tables 1 and 2).

While male surgeons performed a median of 29 cases per quarter (interquartile range 12 to 61), female surgeons performed a median of 13 cases per quarter (interquartile range 7 to 30) (Table 3). Male surgeons’ mode was the 10^th^ decile, with a left-skewed distribution, while female surgeons’ mode was the 2^nd^ decile, with a right-skewed distribution. Suppose that a male and a female surgeons’ quarter were selected randomly. Then, for 66% of such selections (i.e., the c-statistic), the male surgeon would have performed more cases (99% confidence interval 65% to 66%, P < .0001).

**Fig 1:**
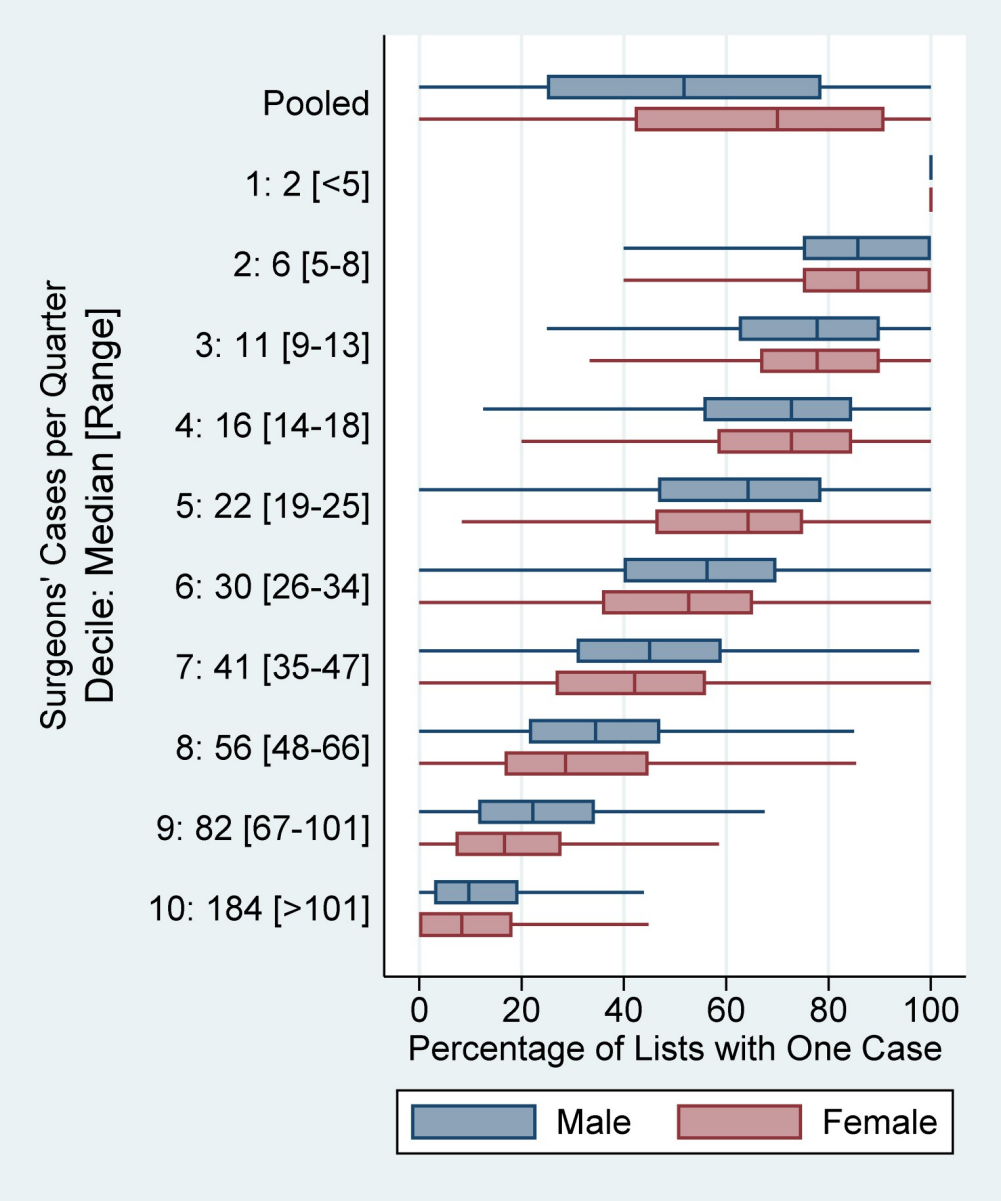
Proportions of surgeon-date-facility combinations (i.e., lists) per quarter with one case, plotted by surgeon gender (colors) and by deciles among surgeons in their cases per quarter (rows). The pooled rows include all surgeon-quarter combinations with at least one case. Those pooled rows show the findings of Hypothesis 1. While 44.2% of male surgeons’ lists of cases had one case, 54.6% of female surgeons’ lists had one case, significantly more (P < .0001). The next 10 rows show the findings of Hypothesis 2. With adjustment for quarterly operative caseloads, the gender association (male < surgeon) is mitigated fully, because male surgeons’ mode was the 10^th^ decile while female surgeons’ mode was the 2^nd^ decile (i.e., they performed fewer cases). Put another way, select at random a male surgeon-quarter and a female surgeon-quarter, both in the same decile of quarterly caseload. The paired surgeons perform surgery on average for the same numbers of workdays to complete those cases. That is what the operating room manager controls and that influences surgeon productivity. For inferential results controlling for specialty see Table 4 and Fig 2. The potential interaction included in the statistical model is shown by the medians of male and female surgeons matching for the lower (e.g., 1^st^ and 2^nd^) deciles but not for the upper (e.g., 9^th^ and 10^th^) deciles.

While 44.2% of male surgeons’ lists of cases (i.e., surgeon-date-facility combination) had one case, 54.6% of female surgeons’ lists had one case (Tables 3 and 4, P < .0001; Fig 1, top row). The value of 54.6% was significantly greater than half (P < .0001). While 65.8% of male surgeons’ lists had one or two cases, 74.8% of female surgeons’ lists had one or two cases (Tables 3 and 4, P < .0001).

**Table 4:** Contrasts between male and female surgeons, reported as mean difference of percentages, 99% confidence interval, and two-sided P-value, N = 1,509,190 lists.

Contrast, Female surgeons minus male surgeons	One case	One or two cases
Gender^a^	10.4%, 10.1% to 10.7%, P < 0.0001	9.1%, 8.8% to 9.3%, P < 0.0001
Gender, Decile of surgeon’s cases during quarter, ^b^ Interaction between gender and decile, surgeon’s specialty, and quarter, with clustering by facility^a^	-2.1%,-4.6% to 0.4%, P = 0.03	-1.8%,-4.1% to 0.5%, P = 0.05
Gender, Decile of surgeon’s cases during quarter, Interaction between gender and decile, surgeon’s specialty, and quarter	-2.1%,-2.5% to -1.7%, P < 0.0001	-1.8%,-2.2% to -1.4%, P < 0.0001
Gender, Decile of surgeon’s cases during quarter, surgeon’s specialty, and quarter, with clustering by facility	-2.8%,-4.5% to -1.0%, P < 0.0001	-2.5%,-5.0% to -0.1%, P = 0.0080
Gender, Decile of surgeon’s cases during quarter, and surgeon’s specialty, with clustering by facility	-2.8%,-4.6% to -1.0%, P < 0.0001	-2.6%,-5.0% to -0.1%, P = 0.0073
Gender, Decile of surgeon’s cases during quarter, with clustering by facility ^a^	-2.3%,-3.9% to -0.7%, P = 0.0002	-3.5%,-5.5% to -1.5%, P < 0.0001
Gender, Decile of surgeon’s cases during quarter	-2.3%,-2.6% to -1.9%, P < 0.0001	-3.5%,-3.8% to -3.2%,P < 0.0001
Gender and surgeon’s specialty, with clustering by facility	4.5%, 2.3% to 6.7%, P < 0.0001	4.5% 2.0% to 7.1%, P < 0.0001

Odds ratios for gender and the covariates (compared to reference categories) are given in the supplemental content, listed in the sequence of the table.

^a^ These six listed values are those shown graphically in Fig 1.

^b^ Deciles are given in Tables 2 and 3.

### Hypothesis 1: Female surgeons have a greater fraction of their lists with only one case

More lists of female surgeons than male surgeons comprised one case (10.4%, P < .0001; Fig 1 top row). Therefore, hypothesis #1 was not rejected. The same result held for lists with one or two cases (9.1%, P < .0001). Both two-sided lower 99% confidence limits exceeded 5.0%, showing managerially important effect sizes.

### Hypothesis 2: Gender differences in fractions of lists with only one case persist after incorporating confounders

**Fig 2:**
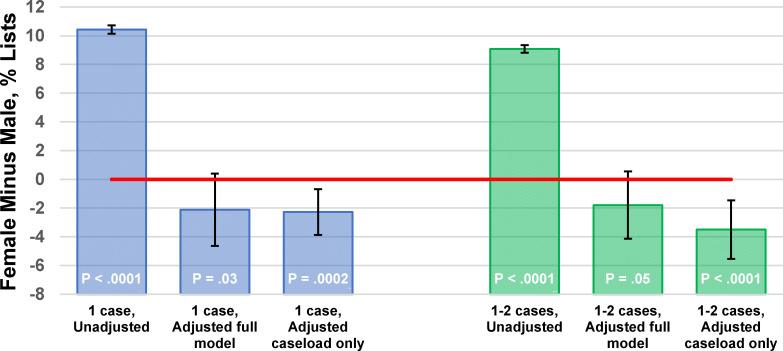
Predictive margins for difference between female surgeons and male surgeons in the percentages of lists of cases (same surgeon, day, and facility combination) with one case (blue) and with one or two cases (green). N = 1,509,190 lists. Error bars show two-sided 99% confidence intervals for the differences in these expected values. The Stata commands and output of these logistic regression models are in the supplemental content. The unadjusted models used gender alone as the independent variable, with robust variance estimation to be consistent with the other models. The full adjusted model included gender, decile of surgeon’s cases during the quarter, interaction between gender and caseload, surgeon’s specialty, and quarter (Tables 2 and 3), and was estimated with clustering by facility. The model with adjustment for quarterly operative caseload only excluded the interaction but was estimated with clustering by facility (Table 4). When estimated without clustering the point estimate was the same but the confidence intervals are narrower, ± 0.2% (Table 4). All variables other than gender were handled by using their average estimates over all observations. The figure shows that unadjusted differences between male and female surgeons vanishes when accounting for the effect of the confounding variables, most importantly caseload. See supplemental content for these averages.

After adjustment for covariates (e.g., quarterly operative caseloads and specialty), there were no significant differences between female and male surgeons in the absolute percentage differences of lists of cases that comprised one case (-2.1%, P = .03; Fig 1). Therefore, hypothesis #2 was rejected. The same result held for lists with one or two cases (-1.8%, P = .05) (Table 4 and Fig 2). The reversals of signs of the estimates from differences suggesting less access of female versus male surgeons (10.4% and 9.1%) to the opposite (-2.1% and -1.8%) were attributable to the differences in quarterly operative caseloads between female and male surgeons (Table 4 and Figs 1 and 2).

## Discussion

Operating room managers have a responsibility to ensure that surgeons and other proceduralists have access to operating room time that is based fairly on the medical requirements of their patients and their quarterly caseloads (workloads). Neither incentive programs nor surgical scheduling should be biased, either directly or indirectly, with respect to protected characteristics (e.g., gender, age) of employees and medical staff [22, 34]. The operating room manager has at most negligible influence over a surgeon’s workload during a quarter but does affect how and when those surgical cases are performed. Our managerial epidemiology study shows the generalizability of Yesantharao et al.’s earlier study of three hospitals to the hundreds of hospitals and ambulatory surgery centers throughout a state [21]. Just like they found, our results (hypothesis #1) showed that surgeon gender was associated with differences in surgical case scheduling [21]. Because of significant differences in quarterly caseloads between male and female surgeons [22], hospitals should expect female surgeons to have substantively (>5%) greater incidences of elective surgical days with only one case, or with one or two cases, than male surgeons. Our results (hypothesis #2) also show that such differences between male and female surgeons in their lists (Fig 1 top row) were not due to systematic bias in operating room scheduling (Fig 1 next ten rows). By this, we mean that when total quarterly workload averages three cases per week, whether that would be one day with three cases or three days each with one case does not significantly differ based on surgeon gender. The frequency of surgical lists including one case was explained principally by the surgeons’ quarterly caseloads, a novel finding because examined among thousands of surgeons.

### Addressing needs of low caseload surgeons (i.e., the majority of female surgeons)

Managers need to recognize that female versus male surgeons’ experiences with case scheduling may differ substantively, because surgeons with single cases (i.e., more often female surgeons) less often have first case starts and therefore have reduced personal productivity from greater tardiness of waiting for preceding late running cases to finish (Fig 1) [7, 21, 29]. There are essentially no conditions wherein these surgeons performing single cases should have been allocated individual block time, because they could not fully fill the workday and partial use of a day cannot be estimated sufficiently accurately [23, 28]. Nevertheless, there is much that operating room managers can do to facilitate case scheduling for these low caseload surgeons, often performing a single “to-follow” case [7, 29]. Such interventions may be country dependent, with prior studies being principally from the USA. Managers can ensure there are processes such that those surgeons with one case to be performed can get their case on the operating room schedule into their service’s shared (allocated) time several weeks in advance when the case is without resource constraints (e.g., only can be performed in a specific room). That can be done probabilistically, not assigning the case to a specific room, but rather providing a confirmed date with an expectation that there will be sufficient capacity for the case, but not with a specified start time [33, 35, 36]. If the service’s time fills, and the low-caseload surgeon has a case to schedule, the manager can release the time of another service forecasted to have substantial unused time [33, 37–39]. When feasible, plan a brief gap (e.g., 30 min) in addition to the average turnover time between the estimated end time of the preceding surgeon in the operating theatre and the start time of the surgeon with the single case [40–42]. Adding a brief gap is especially useful if the preceding cases have a substantial probability of finishing at least 1-hour later than scheduled [42–44]. Such a process reduces the amount of time that a surgeon would be in the facility waiting idly for an operating room to be available. Preceding surgical cases finishing late and thereby reducing the surgeon’s productivity happens far more often than delays in the availability of surgeons due to travel disruptions when coming from clinics or from other hospitals [45].

### Limitations

Our finding of absence of gender-based bias in how operating room cases were scheduled does not negate the fundamentally different question as to why female surgeons performed far fewer cases per quarter. For example, throughout Ontario, female surgeons received fewer procedural referrals than male surgeons, principally because male physicians more often referred patients to male surgeons than female surgeons [46]. That we cannot address this different question is mitigated by the fact that factors influencing surgeon referrals rarely are under the purview of operating room managers (i.e., our study addresses systematic bias in operating room scheduling, not systemic gender-based bias such as due to referral patterns) [22].

In addition to surgeons operating in the state of Florida [9, 26], the observation of overall one or two elective cases per week (Table 2) has similarly been found in multiple previous managerial epidemiology studies from the USA, including three University hospitals [47–49], a large community hospital [50], a city wide health system [29], statewide in Iowa [8, 25], and statewide in Florida among pain medicine physicians’ operative cases [33]. Although suggesting generalizability and the importance of studying surgeons performing single cases on their operative days, our results show that findings may differ among countries depending on surgeons’ average caseloads. Because we studied major therapeutic and major diagnostic procedures (i.e., cases requiring general anesthesia or major conduction blocks) (Table 1), results may differ for countries with surgeons performing proportionately less time evaluating patients, performing office-based procedures, and/or performing minor therapeutic and diagnostic procedures.

We used two years of data (ending just before the start of the COVID-19 pandemic) to achieve a contemporaneous cross-sectional analysis. Had we increased the months of data (or even, potentially, the numbers of surgeons), neither would have substantively reduced our confidence interval widths because these were limited, principally, by the numbers of facilities (Table 4 and Fig 1). In Florida, the number of hospitals does not change markedly from year to year. We studied all non-federal hospitals and all ambulatory surgery centers statewide in Florida. Therefore, greater precision would be obtained by adding more state(s). By doing so, it might be possible to determine if the marginal P-values suggesting possible bias favoring female surgeons is a reliable finding. However, we doubt that such knowledge would be of substantive value because, in retrospect, the practical use would be for the above guidance to the individual operating room manager at single facilities, not to inform statewide or national policy. Furthermore, studying subsets of the Florida data (e.g., by size of facility) to assess potential contributors to bias would be uninformative because of the wide confidence interval widths even when including all data. Thus, we recommend that the focus of additional research related to potential gender bias affecting operating room management should be the referral of operative versus non-operative patients to surgeons differing by gender, as reported from Canada [46]. In other words, based on our findings, we suggest future study to understand better why female surgeons are performing fewer cases per quarter, rather than focusing on how the cases are being scheduled.

The National Provider Identifier database currently only allows providers to self-report a gender of “female” or “male” [30]. Thus, potential influence of other gender identities could not be assessed. However, the prevalence of non-binary surgeons [51] likely is too small to affect our conclusions.

Finally, our paper was limited to data related to individual surgeon productivity and related total growth within operating room budgets [8, 23, 25, 26, 33]. Thus, our paper and results should not be interpreted as having anything to do with operating room nursing or anesthetist efficiency, productivity, utilization, scheduling, or assignment [35–39]. The US administrative state level databases lack case duration data, as needed to analyze such endpoints. However, the latter was not a limitation for our tested hypotheses, because with most surgeons performing one or two cases per day, their individual percentage utilizations could not be measured accurately anyway [23, 28].

## Conclusions

Surgeons’ frequencies of performing one case on operative days were highly dependent on their quarterly caseloads. The differences between male and female surgeons in their lists were not due to systematic bias in operating room scheduling. Thus, health policy planners looking to reduce gender bias related to surgeons’ caseloads should not be focusing on operating room scheduling and managers as potential sources, but rather to external factors such as gender-bias in referrals of cases to surgeons. We found that surgeons performing only one case on their operative (anesthesia) workdays more often were female than male surgeons. This is a previously unrecognized reason why it is important for operating room managers to ease the burden for low caseload surgeons. Improving those surgeons’ access will support better access of female surgeons.

## Supporting information

S1 File Full statistical output from Stata v17.0, including commands and comments, is provided in the supplemental content. These are listed in the same sequence of the Methods and Results for readers interested in more detail or who want to replicate our work with other state or provincial datasets.
(PDF)
