# Supplementary material for: Association of surgeons’ gender with elective surgical lists in the State of Florida is explained by differences in mean operative caseloads
Source: PLoS One. 2023 Mar 15;18(3):e0283033. doi: 10.1371/journal.pone.0283033 (PMC10016664; doi:10.1371/journal.pone.0283033)
Supplement: S1 File — These are listed in the same sequence of the Methods and Results for readers interested in more detail or who want to replicate our work with other state or provincial datasets. (PDF) [file pone.0283033.s001.pdf]

```
version 17.0

cd C:\Users\dexterf\Downloads
use "Surgeon gender Florida v20211021-2031 v3.dta", clear
    describe

* Install boxtid package if not installed
capture which boxtid
if ( _rc ) ssc install boxtid

label define LabelGender 1 "Female" 0 "Male"
label values Gender LabelGender

label define LabelPSI  1 "Cardiothoracic Surgery"  2 "Colorectal Surgery"  3 "Gastroenterology"  4 "General Surgery"    ///
                       5 "Gynecological Oncology"  6 "Neurosurgery"        7 "OB/GYN"   8 "OMFS"   9 "Ophthalmology"      ///
                      10 "Orthopedic Surgery"     11 "Otolaryngology"     12 "Plastic Surgery"  13 "Podiatric Surgery"  ///
                      14 "Surgical Oncology"      15 "Urology"            16 "Vascular Surgery"
                                                  ** Cardiothoracic surgery includes thoracic surgery
                                                  ** General surgery includes transplant, critical care, and pediatric surgery
                                                  ** OMFS includes oral maxillofacial surgery, oral surgery, and dentist
                                                  ** Orthopedic surgery includes hand surgery.
label values PSI LabelPSI

xtile nDecCases = nCases, nq(10)                  // Deciles of number of surgeon cases during the quarter
    by nDecCases, sort : summarize nCases         //    Printout to create labels Mean [range]
    label define LabelnDecCases 1 " 2 [<5]    " 2 " 6 [ 5- 8]" 3 "11 [ 9-13]" 4 "16 [14-18]"    5 "22 [19-25]"  ///
                                6 "30 [26-34]" 7 "41 [35-47]" 8 "56 [48-66]" 9 "82 [67-101]" 10 "184 [>101]"
label values nDecCases LabelnDecCases

save "Stata temp.dta", replace


* Demographics
total nCases nLists                               // Surgeon's number of lists of cases (surgeon-date-facility) during quarter
                                                  // Note, 1509190 lists, the sample size for the logistic regressions below

sort PNPII                                        // National Provider Identifier, blinded
    by PNPII: gen byte nSurg = _N
        assert nSurg > 0 & nSurg < 13             // Confirming, each surgeon has max 12 quarters

    by PNPII: gen nNewSurgeon = (_n == 1)         // https://www.biostat.jhsph.edu/~courses/bio624/misc/sjpdf.pdf#page=6
        count if nNewSurgeon > 0                  // Unique surgeons (e.g., PNPII 2468 at >1 facility counted once)

assert nLists == n1OR2Cases + nGT2Cases
assert n1Case <= n1OR2Cases                       // Number surgeon's lists (surgeon-date-facility) during quarter with 1 case

by FNI, sort: generate nNewFacility = _n == 1     // Facility code, blinded
    count if nNewFacility > 0                     // Number of unique facilities
```

```

tabulate Gender                                  // Number of surgeon quarters, counted separately for female and male surgeons
tabulate Gender [fweight = nNewSurgeon]          // Number of female surgeons and number of male surgeons
tabulate Gender [fweight = nCases      ]         // Cases, counted separately for female and male surgeons
tabulate Gender [fweight = nLists      ]         // Lists, counted separately for female and male surgeons

tabulate nDecCases Gender, row
tabulate nDecCases Gender [fweight = nCases     ], row      // Numbers of cases, will include all quarters
tabulate nDecCases Gender [fweight = nLists     ], row

tabulate PSI Gender, row rowsort                 // Specialties. Below, reference group cardiothoracic surgery simply because
                                                 // that is number "1". Still, that specialty has smallest percentage female
                                                 // by surgeon quarters, surgeons, cases, and lists.
tabulate PSI Gender [fweight = nNewSurgeon], row rowsort
tabulate PSI Gender [fweight = nCases     ], row rowsort
tabulate PSI Gender [fweight = nLists     ], row rowsort

tabulate BinQtr Gender, row                                 // Quarter 01 to 12, Jan 01, 2017 to Dec 31, 2019
tabulate BinQtr Gender [fweight = nCases     ], row
tabulate BinQtr Gender [fweight = nLists     ], row

ratio (One: n1Case/nLists) (OneOrTwo: n1OR2Cases/nLists), level(99) fvwrap(1)
ratio (One: n1Case/nLists) (OneOrTwo: n1OR2Cases/nLists), level(99) fvwrap(1) over(Gender)
ratio (One: n1Case/nLists) (OneOrTwo: n1OR2Cases/nLists), level(99) fvwrap(1) over(nDecCases)
ratio (One: n1Case/nLists) (OneOrTwo: n1OR2Cases/nLists), level(99) fvwrap(1) over(PSI)
ratio (One: n1Case/nLists) (OneOrTwo: n1OR2Cases/nLists), level(99) fvwrap(1) over(BinQtr)
ratio (One: n1Case/nLists) (OneOrTwo: n1OR2Cases/nLists), level(99) fvwrap(1) over(Gender nDecCases)
ratio (One: n1Case/nLists) (OneOrTwo: n1OR2Cases/nLists), level(99) fvwrap(1) over(Gender PSI)

by Gender, sort : summarize nCases, detail                  // Explains results below
    ranksum nCases, by(Gender) porder                       // Interpret Wilcoxon-Mann-Whitney as AUC (WMWodds)
     roctab Gender nCases, binomial level(99)
        display round(100*(1-0.3446),0.1) "%, exact binomial 99% CI " ///
                round(100*(1-0.34862),0.1) ", " round(100*(1-0.34062),0.1)


* Create 20 categories nCases to evaluate functional association with % lists with 1 case
use "Stata temp", clear
sort nCases
xtile n20Cases = nCases, nq(20)
    by n20Cases, sort : egen long Totaln1Case = total(n1Case)
    by n20Cases, sort : egen long TotalLists  = total(nLists)
    by n20Cases, sort : egen double Med20Cases = pctile(nCases), p(50)
drop if n20Cases[_n] == n20Cases[_n+1]
generate double Proportion = round( Totaln1Case / TotalLists, 0.01 )
list n20Cases Totaln1Case TotalLists Med20Cases Proportion, nocompress abbreviate(11) noobs
lowess Proportion Med20Cases, logit              // Nonlinear association with, without logit
lowess Proportion Med20Cases
```

```

*         Reshape the 20 records to 40 rows, one for list has 1 case and one for list has >1 case
*         https://www.stata.com/support/faqs/statistics/logistic-regression-with-grouped-data
generate w0  = TotalLists - Totaln1Case
rename Totaln1Case w1
generate id = _n
reshape long w, i(id) j(y)
    label variable y "1 = List 1 case, 0 = otherwise"
rename w FreqWeights
    label variable FreqWeights "Count of lists, use with fw"

boxtid logit y Med20Cases [fweight = FreqWeights]           // https://FDshort.com/30YXSCm
    display 1/0.1379233                                     // Transform 1/7.25 power; will instead use categories


* Boxplot showing Male/Female without and with category of decile
use "Stata temp", clear
    sort PNPII FNI BinQtr                             // confirming expected structure
    tabulate nDecCases Gender if nLists > 0, column  // males left-skewed mode 10th, females right-skewed mode 2nd

gen double Perc1CaseF = 100* n1Case / nLists if Gender == 1
gen double Perc1CaseM = 100* n1Case / nLists if Gender == 0

* https://statatexblog.com/2019/10/08/create-duplicate-observations-in-stata/
expand 2, gen(dupindicator)
    replace nDecCases = 0 if dupindicator == 1
    label define LabelnDecCasesRev 0 "Pooled"                                                      ///
                                   1 "1: 2 [<5]"       2 "2: 6 [5-8]"    3 "3: 11 [9-13]"  4 "4: 16 [14-18]" ///
                                   5 "5: 22 [19-25]"   6 "6: 30 [26-34]" 7 "7: 41 [35-47]" 8 "8: 56 [48-66]" ///
                                   9 "9: 82 [67-101]" 10 "10: 184 [>101]"
label values nDecCases LabelnDecCasesRev

* https://www.stata.com/support/faqs/graphics/gph/graphdocs/box-plot-of-two-variables-by-categorical-variable/index.html
graph hbox Perc1CaseM Perc1CaseF, over(nDecCases) nooutsides note("") legend(label(1 "Male") label(2 "Female"))  ///
           l2title(Surgeons' Cases per Quarter) l1title(Decile: Median [Range])                                ///
           ytitle(Percentage of Lists with One Case) xsize(5) ysize(6) alsize(0)


* Reshape to two rows per surgeon-quarter, following preceding Stata FAQ link
use "Stata temp", clear

generate w0  = nLists - n1Case
rename n1Case w1
generate id = _n
reshape long w, i(id) j(y)
    label variable y "1 = List 1 case, 0 = otherwise"
rename w FreqWeights
    label variable FreqWeights "Count of lists, use with fw"
```

```

* Listed in sequence of second column of Table 4, top to bottom
logistic y i.Gender [fweight = FreqWeights], vce(robust) level(99)
    margins   Gender, atmeans level(99)
    margins r.Gender, atmeans level(99)          // https://www.stata.com/manuals13/rmarginscontrast.pdf#page=4
        display "Absolute risk difference 0.1043141 equals 0.5460821 - 0.441768"
logistic y i.Gender##i.nDecCases i.PSI i.BinQtr [fweight = FreqWeights], vce(cluster FNI) level(99)
    margins r.Gender, atmeans level(99)
logistic y i.Gender##i.nDecCases i.PSI i.BinQtr [fweight = FreqWeights], vce(robust)      level(99)
    margins r.Gender, atmeans level(99)
logistic y i.Gender  i.nDecCases i.PSI i.BinQtr [fweight = FreqWeights], vce(cluster FNI) level(99)
    margins r.Gender, atmeans level(99)
logistic y i.Gender  i.nDecCases i.PSI          [fweight = FreqWeights], vce(cluster FNI) level(99)
    margins r.Gender, atmeans level(99)
logistic y i.Gender  i.nDecCases                [fweight = FreqWeights], vce(cluster FNI) level(99)
    margins r.Gender, atmeans level(99)
logistic y i.Gender  i.nDecCases                [fweight = FreqWeights], vce(robust)      level(99)
    margins r.Gender, atmeans level(99)
logistic y i.Gender              i.PSI          [fweight = FreqWeights], vce(cluster FNI) level(99)
    margins r.Gender, atmeans level(99)


* Sensitivity analysis using 1 or 2 cases instead of 1 case
use "Stata temp", clear

generate w0  = nLists - n1OR2Cases
rename n1OR2Cases w1
generate id = _n
reshape long w, i(id) j(y)
    label variable y "1 = List 1 or 2 cases, 0 = otherwise"
rename w FreqWeights
    label variable FreqWeights "Count of lists, use with fw"

* Listed in sequence of third column of Table 4, top to bottom
logistic y i.Gender [fweight = FreqWeights], vce(robust) level(99)
    margins r.Gender, atmeans level(99)
logistic y i.Gender##i.nDecCases i.PSI i.BinQtr [fweight = FreqWeights], vce(cluster FNI) level(99)
    margins r.Gender, atmeans level(99)
logistic y i.Gender##i.nDecCases i.PSI i.BinQtr [fweight = FreqWeights], vce(robust)      level(99)
    margins r.Gender, atmeans level(99)
logistic y i.Gender  i.nDecCases i.PSI i.BinQtr [fweight = FreqWeights], vce(cluster FNI) level(99)
    margins r.Gender, atmeans level(99)
logistic y i.Gender  i.nDecCases i.PSI          [fweight = FreqWeights], vce(cluster FNI) level(99)
    margins r.Gender, atmeans level(99)
logistic y i.Gender  i.nDecCases                [fweight = FreqWeights], vce(cluster FNI) level(99)
    margins r.Gender, atmeans level(99)
logistic y i.Gender  i.nDecCases                [fweight = FreqWeights], vce(robust)      level(99)
    margins r.Gender, atmeans level(99)
```

```
logistic y i.Gender             i.PSI           [fweight = FreqWeights], vce(cluster FNI) level(99)
    margins r.Gender, atmeans level(99)
```

```
. use "Surgeon gender Florida v20211021-2031 v3.dta", clear

.         describe

Contains data from Surgeon gender Florida v20211021-2031 v3.dta
 Observations:        94,005
    Variables:            12                  25 Nov 2021 19:50
--------------------------------------------------------------------------------
Variable      Storage   Display    Value
    name         type    format    label      Variable label
--------------------------------------------------------------------------------
PNPII           int     %10.0g                Performing NPI Index
Gender          byte    %10.0g              * 0 Male 1 Female
PSI             byte    %10.0g                Primary Specialty Index
GradYear        int     %10.0g                medical school graduation year
FNI             int     %10.0g                facility number index
BinQtr          byte    %10.0g                Quarter of the year from Jan 01 2017
nDaysOperating  byte    %10.0g                Days the surgeon operated during the quarter
nCases          int     %10.0g                Cases the surgeon performed during the quarter
n1Case          byte    %10.0g                Lists in the quarter with 1 case, Lists facility x surgeon x date
n1OR2Cases      byte    %10.0g                Lists in the quarter with 1 or 2 cases, Lists facility x surgeon x date
nGT2Cases       byte    %10.0g                Lists in the quarter with >2 cases
nLists          byte    %10.0g                Lists in the quarter, total
                                            * indicated variables have notes
--------------------------------------------------------------------------------
Sorted by:

.
. * Install boxtid package if not installed
. capture which boxtid

. if ( _rc ) ssc install boxtid

.
. label define LabelGender 1 "Female" 0 "Male"

. label values Gender LabelGender

.
. label define LabelPSI  1 "Cardiothoracic Surgery"  2 "Colorectal Surgery"  3 "Gastroenterology"  4 "General Surgery"    ///
>                        5 "Gynecological Oncology"  6 "Neurosurgery"        7 "OB/GYN"  8 "OMFS"  9 "Ophthalmology"      ///
>                       10 "Orthopedic Surgery"     11 "Otolaryngology"     12 "Plastic Surgery"  13 "Podiatric Surgery"  ///
>                       14 "Surgical Oncology"      15 "Urology"            16 "Vascular Surgery"
```

```
.                                                    ** Cardiothoracic surgery includes thoracic surgery
.                                                    ** General surgery includes transplant, critical care, and pediatric surgery
.                                                    ** OMFS includes oral maxillofacial surgery, oral surgery, and dentist
.                                                    ** Orthopedic surgery includes hand surgery.
. label values PSI LabelPSI

.
. xtile nDecCases = nCases, nq(10)                  // Deciles of number of surgeon cases during the quarter

.         by nDecCases, sort : summarize nCases         //        Printout to create labels Mean [range]
```

-> nDecCases = 1

Variable	Obs	Mean	Std. dev.	Min	Max
nCases	9,526	2.492127	1.360828	0	4

-> nDecCases = 2

Variable	Obs	Mean	Std. dev.	Min	Max
nCases	9,866	6.452463	1.121246	5	8

-> nDecCases = 3

Variable	Obs	Mean	Std. dev.	Min	Max
nCases	10,574	10.92065	1.420367	9	13

-> nDecCases = 4

Variable	Obs	Mean	Std. dev.	Min	Max
nCases	8,651	15.93619	1.418816	14	18

-> nDecCases = 5

Variable	Obs	Mean	Std. dev.	Min	Max
nCases	9,462	21.80268	1.996525	19	25

-> nDecCases = 6

Variable	Obs	Mean	Std. dev.	Min	Max
nCases	9,043	29.80383	2.615185	26	34

-> nDecCases = 7

Variable	Obs	Mean	Std. dev.	Min	Max
nCases	9,249	40.64861	3.7148	35	47

-> nDecCases = 8

Variable	Obs	Mean	Std. dev.	Min	Max
nCases	9,082	56.27054	5.467731	48	66

```
-> nDecCases = 9

    Variable |        Obs        Mean    Std. dev.       Min        Max
-------------+---------------------------------------------------------
      nCases |      9,212    81.86138    9.997068         67        101

-------------------------------------------------------------------------------------------------------------------------------
-> nDecCases = 10

    Variable |        Obs        Mean    Std. dev.       Min        Max
-------------+---------------------------------------------------------
      nCases |      9,340    184.0347    123.6189        102       1439


.         label define LabelnDecCases 1 " 2 [<5]   " 2 " 6 [ 5- 8]" 3 "11 [ 9-13]" 4 "16 [14-18]"   5 "22 [19-25]"  ///
>                                 6 "30 [26-34]" 7 "41 [35-47]" 8 "56 [48-66]" 9 "82 [67-101]" 10 "184 [>101]"

. label values nDecCases LabelnDecCases

.
. save "Stata temp.dta", replace
file Stata temp.dta saved

.
.
. * Demographics
. total nCases nLists                                                                       // Surgeon's number of lists of cases (surgeon-date-facility) during quarter

Total estimation                        Number of obs = 94,005

--------------------------------------------------------------
             |      Total   Std. err.     [95% conf. interval]
-------------+------------------------------------------------
      nCases |    4176551    19930.17       4137488     4215614
      nLists |    1509190    3281.785       1502758     1515622
--------------------------------------------------------------

.                                                                                                         // Note, 1509190 lists, the sample size for the logistic regressions below
.
. sort PNPII                                                                                      // National Provider Identifier, blinded

.         by PNPII: gen byte nSurg = _N

.                 assert nSurg > 0 & nSurg < 13                 // Confirming, each surgeon has max 12 quarters

.
.         by PNPII: gen nNewSurgeon = (_n == 1)         // https://www.biostat.jhsph.edu/~courses/bio624/misc/sjpdf.pdf#page=6

.                 count if nNewSurgeon > 0                          // Unique surgeons (e.g., PNPII 2468 at >1 facility counted once)
  8,875

.
. assert nLists == n1OR2Cases + nGT2Cases

. assert n1Case <= n1OR2Cases                       // Number surgeon's lists (surgeon-date-facility) during quarter with 1 case
```

```
.
. by FNI, sort: generate nNewFacility = _n == 1     // Facility code, blinded

.         count if nNewFacility > 0                         // Number of unique facilities
  609

.
. tabulate Gender                                                           // Number of surgeon quarters, counted separately for female and male surgeons
```

0 Male 1 Female	Freq.	Percent	Cum.
Male	75,382	80.19	80.19
Female	18,623	19.81	100.00
Total	94,005	100.00	

```
. tabulate Gender [fweight = nNewSurgeon]                   // Number of female surgeons and number of male surgeons
```

0 Male 1 Female	Freq.	Percent	Cum.
Male	7,045	79.38	79.38
Female	1,830	20.62	100.00
Total	8,875	100.00	

```
. tabulate Gender [fweight = nCases     ]           // Cases, counted separately for female and male surgeons
```

0 Male 1 Female	Freq.	Percent	Cum.
Male	3,655,477	87.52	87.52
Female	521,074	12.48	100.00
Total	4,176,551	100.00	

```
. tabulate Gender [fweight = nLists     ]           // Lists, counted separately for female and male surgeons
```

0 Male 1 Female	Freq.	Percent	Cum.
Male	1,291,893	85.60	85.60
Female	217,297	14.40	100.00
Total	1,509,190	100.00	

```
.
. tabulate nDecCases Gender, row
```

Key
frequency<br>*row percentage*

10 quantiles of nCases	0 Male 1 Female Male	Female	Total
2 [<5]	**6,636**<br>**69.66**	**2,890**<br>**30.34**	**9,526**<br>**100.00**
6 [5- 8]	**6,558**<br>**66.47**	**3,308**<br>**33.53**	**9,866**<br>**100.00**
11 [9-13]	**7,308**<br>**69.11**	**3,266**<br>**30.89**	**10,574**<br>**100.00**
16 [14-18]	**6,584**<br>**76.11**	**2,067**<br>**23.89**	**8,651**<br>**100.00**
22 [19-25]	**7,713**<br>**81.52**	**1,749**<br>**18.48**	**9,462**<br>**100.00**
30 [26-34]	**7,772**<br>**85.94**	**1,271**<br>**14.06**	**9,043**<br>**100.00**
41 [35-47]	**8,075**<br>**87.31**	**1,174**<br>**12.69**	**9,249**<br>**100.00**
56 [48-66]	**7,980**<br>**87.87**	**1,102**<br>**12.13**	**9,082**<br>**100.00**
82 [67-101]	**8,291**<br>**90.00**	**921**<br>**10.00**	**9,212**<br>**100.00**
184 [>101]	**8,465**<br>**90.63**	**875**<br>**9.37**	**9,340**<br>**100.00**
Total	**75,382**<br>**80.19**	**18,623**<br>**19.81**	**94,005**<br>**100.00**

```
. tabulate nDecCases Gender [fweight = nCases     ], row      // Numbers of cases, will include all quarters
```

Key
frequency<br>*row percentage*

10 quantiles of nCases	0 Male 1 Female Male	Female	Total
2 [<5]	**16,415**<br>**69.14**	**7,325**<br>**30.86**	**23,740**<br>**100.00**
6 [5- 8]	**42,375**<br>**66.56**	**21,285**<br>**33.44**	**63,660**<br>**100.00**
11 [9-13]	**79,931**<br>**69.22**	**35,544**<br>**30.78**	**115,475**<br>**100.00**
16 [14-18]	**105,031**<br>**76.18**	**32,833**<br>**23.82**	**137,864**<br>**100.00**
22 [19-25]	**168,474**<br>**81.67**	**37,823**<br>**18.33**	**206,297**<br>**100.00**
30 [26-34]	**231,875**<br>**86.03**	**37,641**<br>**13.97**	**269,516**<br>**100.00**

41 [35-47]	328,444<br>87.36	47,515<br>12.64	375,959<br>100.00
56 [48-66]	449,217<br>87.90	61,832<br>12.10	511,049<br>100.00
82 [67-101]	678,215<br>89.94	75,892<br>10.06	754,107<br>100.00
184 [>101]	1,555,500<br>90.49	163,384<br>9.51	1,718,884<br>100.00
Total	3,655,477<br>87.52	521,074<br>12.48	4,176,551<br>100.00

```
. tabulate nDecCases Gender [fweight = nLists     ], row
```

Key
frequency<br>*row percentage*

10 quantiles of nCases	0 Male 1 Female<br>Male	 Female	Total
2 [<5]	15,333<br>69.01	6,886<br>30.99	22,219<br>100.00
6 [5- 8]	36,147<br>66.39	18,302<br>33.61	54,449<br>100.00
11 [9-13]	62,870<br>69.07	28,154<br>30.93	91,024<br>100.00
16 [14-18]	76,584<br>75.91	24,302<br>24.09	100,886<br>100.00
22 [19-25]	113,320<br>81.90	25,048<br>18.10	138,368<br>100.00
30 [26-34]	140,796<br>86.57	21,851<br>13.43	162,647<br>100.00
41 [35-47]	173,625<br>87.92	23,849<br>12.08	197,474<br>100.00
56 [48-66]	199,216<br>88.66	25,488<br>11.34	224,704<br>100.00
82 [67-101]	232,301<br>91.17	22,486<br>8.83	254,787<br>100.00
184 [>101]	241,701<br>92.03	20,931<br>7.97	262,632<br>100.00
Total	1,291,893<br>85.60	217,297<br>14.40	1,509,190<br>100.00

```
.
. tabulate PSI Gender, row rowsort                              // Specialties. Below, reference group cardiothoracic surgery simply because
```

Key
frequency   *row percentage*

Primary Specialty Index	0 Male 1 Female Male	Female	Total
OB/GYN	9,252<br>50.06	9,228<br>49.94	18,480<br>100.00
Orthopedic Surgery	15,046<br>94.77	830<br>5.23	15,876<br>100.00
General Surgery	9,662<br>83.46	1,915<br>16.54	11,577<br>100.00
Ophthalmology	8,967<br>79.96	2,247<br>20.04	11,214<br>100.00
Urology	6,765<br>95.43	324<br>4.57	7,089<br>100.00
Podiatric Surgery	5,426<br>80.88	1,283<br>19.12	6,709<br>100.00
Otolaryngology	4,951<br>85.78	821<br>14.22	5,772<br>100.00
Plastic Surgery	3,802<br>86.02	618<br>13.98	4,420<br>100.00
Neurosurgery	3,594<br>94.11	225<br>5.89	3,819<br>100.00
Cardiothoracic Surger	2,638<br>97.24	75<br>2.76	2,713<br>100.00
Vascular Surgery	1,905<br>90.33	204<br>9.67	2,109<br>100.00
Colorectal Surgery	1,034<br>81.61	233<br>18.39	1,267<br>100.00
OMFS	851<br>93.31	61<br>6.69	912<br>100.00
Gynecological Oncolog	446<br>58.92	311<br>41.08	757<br>100.00
Surgical Oncology	472<br>72.84	176<br>27.16	648<br>100.00
Gastroenterology	571<br>88.80	72<br>11.20	643<br>100.00
Total	75,382<br>80.19	18,623<br>19.81	94,005<br>100.00

```
.                                                   // that is number "1". Still, that specialty has smallest percentage female
.                                                   // by surgeon quarters, surgeons, cases, and lists.
. tabulate PSI Gender [fweight = nNewSurgeon], row rowsort
```

Key
frequency<br>*row percentage*

Primary Specialty Index	0 Male 1 Female Male	Female	Total
OB/GYN	853<br>48.55	904<br>51.45	1,757<br>100.00
Orthopedic Surgery	1,381<br>94.27	84<br>5.73	1,465<br>100.00
General Surgery	914<br>82.94	188<br>17.06	1,102<br>100.00
Ophthalmology	816<br>79.69	208<br>20.31	1,024<br>100.00
Urology	629<br>95.16	32<br>4.84	661<br>100.00
Podiatric Surgery	519<br>79.85	131<br>20.15	650<br>100.00
Otolaryngology	461<br>85.53	78<br>14.47	539<br>100.00
Plastic Surgery	357<br>84.80	64<br>15.20	421<br>100.00
Neurosurgery	337<br>93.09	25<br>6.91	362<br>100.00
Cardiothoracic Surger	260<br>96.30	10<br>3.70	270<br>100.00
Vascular Surgery	176<br>88.89	22<br>11.11	198<br>100.00
Colorectal Surgery	100<br>81.97	22<br>18.03	122<br>100.00
OMFS	90<br>92.78	7<br>7.22	97<br>100.00
Gastroenterology	69<br>89.61	8<br>10.39	77<br>100.00
Gynecological Oncolog	40<br>57.97	29<br>42.03	69<br>100.00
Surgical Oncology	43<br>70.49	18<br>29.51	61<br>100.00
Total	7,045<br>79.38	1,830<br>20.62	8,875<br>100.00

. tabulate PSI Gender [fweight = nCases   ], row rowsort

Key
frequency<br>*row percentage*

Primary Specialty Index	0 Male 1 Female Male	Female	Total
Ophthalmology	1,063,839<br>85.61	178,848<br>14.39	1,242,687<br>100.00
Orthopedic Surgery	965,865<br>96.11	39,071<br>3.89	1,004,936<br>100.00
General Surgery	490,598<br>87.00	73,280<br>13.00	563,878<br>100.00
OB/GYN	164,189<br>60.41	107,594<br>39.59	271,783<br>100.00
Otolaryngology	199,810<br>82.58	42,136<br>17.42	241,946<br>100.00
Urology	194,384<br>94.57	11,161<br>5.43	205,545<br>100.00
Plastic Surgery	131,734<br>89.25	15,872<br>10.75	147,606<br>100.00
Neurosurgery	134,933<br>96.84	4,403<br>3.16	139,336<br>100.00
Podiatric Surgery	79,464<br>87.69	11,157<br>12.31	90,621<br>100.00
Colorectal Surgery	52,078<br>82.37	11,144<br>17.63	63,222<br>100.00
Cardiothoracic Surger	57,971<br>98.44	917<br>1.56	58,888<br>100.00
Vascular Surgery	54,089<br>92.88	4,144<br>7.12	58,233<br>100.00
Gynecological Oncolog	27,407<br>67.78	13,028<br>32.22	40,435<br>100.00
Surgical Oncology	21,905<br>75.80	6,994<br>24.20	28,899<br>100.00
OMFS	10,909<br>94.83	595<br>5.17	11,504<br>100.00
Gastroenterology	6,302<br>89.62	730<br>10.38	7,032<br>100.00
Total	3,655,477<br>87.52	521,074<br>12.48	4,176,551<br>100.00

. tabulate PSI Gender [fweight = nLists   ], row rowsort

Key
frequency
row percentage

Primary Specialty Index	0 Male 1 Female Male	Female	Total
Orthopedic Surgery	**326,015**<br>**95.63**	**14,906**<br>**4.37**	**340,921**<br>**100.00**
General Surgery	**222,891**<br>**86.25**	**35,545**<br>**13.75**	**258,436**<br>**100.00**
OB/GYN	**112,671**<br>**58.63**	**79,505**<br>**41.37**	**192,176**<br>**100.00**
Ophthalmology	**135,335**<br>**82.13**	**29,452**<br>**17.87**	**164,787**<br>**100.00**
Urology	**103,399**<br>**95.53**	**4,843**<br>**4.47**	**108,242**<br>**100.00**
Otolaryngology	**75,983**<br>**85.84**	**12,529**<br>**14.16**	**88,512**<br>**100.00**
Plastic Surgery	**64,917**<br>**86.94**	**9,755**<br>**13.06**	**74,672**<br>**100.00**
Neurosurgery	**68,983**<br>**95.89**	**2,955**<br>**4.11**	**71,938**<br>**100.00**
Podiatric Surgery	**48,123**<br>**86.18**	**7,718**<br>**13.82**	**55,841**<br>**100.00**
Cardiothoracic Surger	**43,350**<br>**98.07**	**852**<br>**1.93**	**44,202**<br>**100.00**
Vascular Surgery	**31,765**<br>**91.79**	**2,840**<br>**8.21**	**34,605**<br>**100.00**
Colorectal Surgery	**25,891**<br>**82.59**	**5,459**<br>**17.41**	**31,350**<br>**100.00**
Gynecological Oncolog	**10,781**<br>**63.24**	**6,267**<br>**36.76**	**17,048**<br>**100.00**
Surgical Oncology	**10,967**<br>**75.12**	**3,633**<br>**24.88**	**14,600**<br>**100.00**
OMFS	**6,987**<br>**93.82**	**460**<br>**6.18**	**7,447**<br>**100.00**
Gastroenterology	**3,835**<br>**86.90**	**578**<br>**13.10**	**4,413**<br>**100.00**
Total	**1,291,893**<br>**85.60**	**217,297**<br>**14.40**	**1,509,190**<br>**100.00**

.
. tabulate BinQtr Gender, row   // Quarter 01 to 12, Jan 01, 2017 to Dec 31, 2019

Key
frequency   *row percentage*

Quarter of the year from Jan 01 2017	0 Male 1 Female Male	 Female	Total
1	**5,891** **81.51**	**1,336** **18.49**	**7,227** **100.00**
2	**6,126** **80.88**	**1,448** **19.12**	**7,574** **100.00**
3	**6,313** **80.66**	**1,514** **19.34**	**7,827** **100.00**
4	**6,423** **80.26**	**1,580** **19.74**	**8,003** **100.00**
5	**6,463** **80.23**	**1,593** **19.77**	**8,056** **100.00**
6	**6,458** **80.22**	**1,592** **19.78**	**8,050** **100.00**
7	**6,442** **80.03**	**1,607** **19.97**	**8,049** **100.00**
8	**6,487** **79.69**	**1,653** **20.31**	**8,140** **100.00**
9	**6,417** **79.55**	**1,650** **20.45**	**8,067** **100.00**
10	**6,340** **79.56**	**1,629** **20.44**	**7,969** **100.00**
11	**6,142** **79.72**	**1,562** **20.28**	**7,704** **100.00**
12	**5,880** **80.12**	**1,459** **19.88**	**7,339** **100.00**
Total	**75,382** **80.19**	**18,623** **19.81**	**94,005** **100.00**

. tabulate BinQtr Gender [fweight = nCases   ], row

Key
frequency   *row percentage*

Quarter of the year from Jan 01 2017	0 Male 1 Female Male	Female	Total
1	307,288<br>88.38	40,411<br>11.62	347,699<br>100.00
2	306,634<br>87.97	41,940<br>12.03	348,574<br>100.00
3	274,875<br>87.82	38,109<br>12.18	312,984<br>100.00
4	308,028<br>87.97	42,134<br>12.03	350,162<br>100.00
5	309,646<br>87.75	43,244<br>12.25	352,890<br>100.00
6	313,597<br>87.63	44,248<br>12.37	357,845<br>100.00
7	296,658<br>87.28	43,250<br>12.72	339,908<br>100.00
8	309,418<br>87.28	45,097<br>12.72	354,515<br>100.00
9	307,787<br>87.21	45,127<br>12.79	352,914<br>100.00
10	315,648<br>87.12	46,660<br>12.88	362,308<br>100.00
11	298,537<br>86.73	45,679<br>13.27	344,216<br>100.00
12	307,361<br>87.19	45,175<br>12.81	352,536<br>100.00
Total	3,655,477<br>87.52	521,074<br>12.48	4,176,551<br>100.00

```
. tabulate BinQtr Gender [fweight = nLists     ], row
```

Key
frequency<br>*row percentage*

Quarter of the year from Jan 01 2017	0 Male 1 Female Male	Female	Total
1	108,977<br>86.61	16,854<br>13.39	125,831<br>100.00
2	110,265<br>86.34	17,440<br>13.66	127,705<br>100.00
3	100,952<br>86.15	16,234<br>13.85	117,186<br>100.00
4	109,620	17,743	127,363

	86.07	13.93	100.00
5	109,773<br>85.96	17,931<br>14.04	127,704<br>100.00
6	110,573<br>85.79	18,315<br>14.21	128,888<br>100.00
7	105,612<br>85.29	18,217<br>14.71	123,829<br>100.00
8	108,818<br>85.16	18,968<br>14.84	127,786<br>100.00
9	107,320<br>85.08	18,817<br>14.92	126,137<br>100.00
10	110,403<br>85.11	19,315<br>14.89	129,718<br>100.00
11	104,910<br>84.66	19,015<br>15.34	123,925<br>100.00
12	104,670<br>85.02	18,448<br>14.98	123,118<br>100.00
Total	1,291,893<br>85.60	217,297<br>14.40	1,509,190<br>100.00

```
.
. ratio (One: n1Case/nLists) (OneOrTwo: n1OR2Cases/nLists), level(99) fvwrap(1)
```

Ratio estimation   Number of obs = **94,005**

One: **n1Case/nLists**
OneOrTwo: **n1OR2Cases/nLists**

	Ratio	Linearized std. err.	[99% conf.	interval]
One	.4567874	.00102	.4541601	.4594147
OneOrTwo	.6708069	.0011505	.6678434	.6737703

```
. ratio (One: n1Case/nLists) (OneOrTwo: n1OR2Cases/nLists), level(99) fvwrap(1) over(Gender)
```

Ratio estimation   Number of obs = **94,005**

One: **n1Case/nLists**
OneOrTwo: **n1OR2Cases/nLists**

	Ratio	Linearized std. err.	[99% conf.	interval]
c.One@Gender				
Male	.441768	.0011026	.4389278	.4446083
Female	.5460821	.0025752	.5394487	.5527154
c.OneOrTwo@Gender				
Male	.6577348	.0012626	.6544824	.6609872
Female	.7485239	.0026556	.7416834	.7553644

. ratio (One: n1Case/nLists) (OneOrTwo: n1OR2Cases/nLists), level(99) fvwrap(1) over(nDecCases)

Ratio estimation   Number of obs = **94,005**

One: **n1Case/nLists**
OneOrTwo: **n1OR2Cases/nLists**

	Ratio	Linearized std. err.	[99% conf.	interval]
c.One@nDecCases				
2 [<5]	.9361807	.0016235	.9319988	.9403627
6 [5- 8]	.8553325	.001712	.8509226	.8597424
11 [9-13]	.7865947	.0017424	.7821064	.7910831
16 [14-18]	.7281783	.001995	.7230394	.7333173
22 [19-25]	.6617209	.0020041	.6565586	.6668833
30 [26-34]	.5838103	.0020887	.57843	.5891907
41 [35-47]	.4893708	.0020505	.484089	.4946525
56 [48-66]	.3837849	.0019447	.3787757	.3887941
82 [67-101]	.2693269	.0017632	.2647851	.2738688
184 [>101]	.148234	.0014716	.1444433	.1520248
c.OneOrTwo@nDecCases				
2 [<5]	.9956344	.000443	.9944931	.9967756
6 [5- 8]	.9791915	.0006439	.9775328	.9808502
11 [9-13]	.9562094	.0008075	.9541293	.9582894
16 [14-18]	.9301786	.0010843	.9273855	.9329718
22 [19-25]	.8943903	.0012735	.89111	.8976707
30 [26-34]	.8465081	.0015773	.842445	.8505711
41 [35-47]	.7716408	.0018649	.7668372	.7764445
56 [48-66]	.6596367	.0021272	.6541573	.665116
82 [67-101]	.4959711	.0023927	.4898079	.5021344
184 [>101]	.2575886	.0023481	.25154	.2636371

. ratio (One: n1Case/nLists) (OneOrTwo: n1OR2Cases/nLists), level(99) fvwrap(1) over(PSI)

Ratio estimation   Number of obs = **94,005**

One: **n1Case/nLists**
OneOrTwo: **n1OR2Cases/nLists**

	Ratio	Linearized std. err.	[99% conf.	interval]
c.One@PSI				
Cardiothoracic Surgery	.7374327	.0051513	.7241635	.7507019
Colorectal Surgery	.4814354	.0061766	.4655252	.4973456
Gastroenterology	.6979379	.0168885	.654435	.7414408
General Surgery	.4336083	.0022538	.4278029	.4394138
Gynecological Oncology	.3565228	.0079269	.336104	.3769416
Neurosurgery	.4941199	.0047244	.4819505	.5062894
OB/GYN	.7166139	.0020298	.7113855	.7218423
OMFS	.7122331	.0104167	.6854008	.7390654
Ophthalmology	.1940687	.0020942	.1886744	.199463
Orthopedic Surgery	.3227317	.0018861	.3178732	.3275901
Otolaryngology	.4245187	.0036522	.415111	.4339264
Plastic Surgery	.5494161	.0043656	.5381709	.5606614
Podiatric Surgery	.6314357	.0036737	.6219727	.6408987
Surgical Oncology	.4658219	.0094685	.4414321	.4902118
Urology	.5433104	.0030748	.5353901	.5512306
Vascular Surgery	.5903482	.005557	.5760341	.6046623
c.OneOrTwo@PSI				
Cardiothoracic Surgery	.9427401	.0029457	.9351524	.9503279
Colorectal Surgery	.736874	.0058927	.721695	.752053

Gastroenterology	.8744618	.0144046	.8373572	.9115664
General Surgery	.6826913	.0024957	.6762625	.68912
Gynecological Oncology	.6099249	.0101444	.583794	.6360558
Neurosurgery	.7692179	.0046973	.7571183	.7813175
OB/GYN	.9094684	.0014663	.9056913	.9132455
OMFS	.9094938	.0090824	.8860986	.9328889
Ophthalmology	.3037558	.0028991	.296288	.3112235
Orthopedic Surgery	.5348893	.0024584	.5285568	.5412218
Otolaryngology	.6504768	.0043503	.6392708	.6616827
Plastic Surgery	.7800782	.0043088	.7689793	.7911772
Podiatric Surgery	.8484089	.0029501	.8408097	.856008
Surgical Oncology	.7356164	.0097457	.7105126	.7607203
Urology	.7831341	.0029601	.7755093	.7907589
Vascular Surgery	.8353706	.0049597	.822595	.8481462

```
. ratio (One: n1Case/nLists) (OneOrTwo: n1OR2Cases/nLists), level(99) fvwrap(1) over(BinQtr)
```

Ratio estimation   Number of obs = **94,005**

One: **n1Case/nLists**
OneOrTwo: **n1OR2Cases/nLists**

	Ratio	Linearized std. err.	[99% conf.	interval]
c.One@BinQtr				
1	.4587741	.0036553	.4493584	.4681897
2	.4634509	.0035369	.4543403	.4725615
3	.4694759	.0035373	.4603642	.4785876
4	.4595919	.0034964	.4505856	.4685982
5	.4647936	.0035452	.4556616	.4739256
6	.4596627	.0035153	.4506077	.4687176
7	.4596823	.0035128	.4506337	.4687309
8	.4545255	.0034779	.4455669	.4634842
9	.4548943	.0035482	.4457546	.464034
10	.4513869	.0035352	.4422807	.460493
11	.4459956	.0035043	.4369688	.4550223
12	.4394808	.0035003	.4304646	.4484971
c.OneOrTwo@BinQtr				
1	.6703038	.0041126	.6597102	.6808974
2	.6782976	.0039756	.668057	.6885383
3	.6843565	.0039055	.6742963	.6944167
4	.6710034	.0039152	.6609183	.6810884
5	.6758285	.0039852	.6655629	.686094
6	.6740891	.003966	.6638732	.684305
7	.6744624	.0039561	.664272	.6846527
8	.6676083	.0039195	.6575122	.6777045
9	.6687728	.0040147	.6584315	.6791141
10	.6673785	.0040073	.6570561	.6777008
11	.6642405	.0039829	.6539809	.6745
12	.6537549	.0040401	.6433481	.6641617

```
. ratio (One: n1Case/nLists) (OneOrTwo: n1OR2Cases/nLists), level(99) fvwrap(1) over(Gender nDecCases)
```

Ratio estimation   Number of obs = **94,005**

One: **n1Case/nLists**
OneOrTwo: **n1OR2Cases/nLists**

	Ratio	Linearized std. err.	[99% conf.	interval]
c.One@Gender#nDecCases				
Male# 2 [<5]	.9345203	.0019827	.9294132	.9396274
Male# 6 [5- 8]	.8543448	.0021173	.8488909	.8597986
Male#11 [9-13]	.7863687	.0021247	.7808958	.7918416
Male#16 [14-18]	.7268881	.0023274	.7208929	.7328833
Male#22 [19-25]	.6652312	.0022283	.6594914	.670971
Male#30 [26-34]	.5888662	.0022464	.5830796	.5946527
Male#41 [35-47]	.492527	.0021881	.4868907	.4981633
Male#56 [48-66]	.3875542	.0020605	.3822466	.3928618
Male#82 [67-101]	.2732834	.0018537	.2685083	.2780584
Male#184 [>101]	.1485265	.0015383	.1445641	.1524889
Female# 2 [<5]	.939878	.0028201	.9326137	.9471424
Female# 6 [5- 8]	.8572834	.0029083	.8497918	.8647749
Female#11 [9-13]	.7870995	.0030379	.7792742	.7949249
Female#16 [14-18]	.7322443	.0038497	.7223279	.7421606
Female#22 [19-25]	.64584	.0045457	.6341307	.6575493
Female#30 [26-34]	.5512334	.0055691	.5368879	.5655788
Female#41 [35-47]	.4663927	.0058238	.4513913	.4813942
Female#56 [48-66]	.3543236	.005809	.3393604	.3692868
Female#82 [67-101]	.2284533	.0054928	.2143043	.2426022
Female#184 [>101]	.1448569	.0050488	.1318519	.157862
c.OneOrTwo@Gender#nDecCases				
Male# 2 [<5]	.9953042	.0005532	.9938793	.9967292
Male# 6 [5- 8]	.9778128	.0008231	.9756927	.979933
Male#11 [9-13]	.9547479	.0010033	.9521636	.9573322
Male#16 [14-18]	.9286274	.0012726	.9253494	.9319054
Male#22 [19-25]	.8957642	.0014135	.8921232	.8994052
Male#30 [26-34]	.8502869	.0016834	.8459508	.8546231
Male#41 [35-47]	.7762131	.0019811	.7711099	.7813163
Male#56 [48-66]	.6652729	.0022403	.6595021	.6710436
Male#82 [67-101]	.5024214	.0024989	.4959846	.5088582
Male#184 [>101]	.259548	.0024582	.2532158	.2658801
Female# 2 [<5]	.9963694	.0007256	.9945003	.9982386
Female# 6 [5- 8]	.9819145	.0010141	.9793024	.9845267
Female#11 [9-13]	.9594729	.0013411	.9560185	.9629273
Female#16 [14-18]	.9350671	.0020464	.9297957	.9403385
Female#22 [19-25]	.8881747	.0029235	.880644	.8957054
Female#30 [26-34]	.8221592	.0044272	.8107551	.8335632
Female#41 [35-47]	.7383538	.0054116	.7244142	.7522934
Female#56 [48-66]	.6155838	.0065811	.5986317	.6325359
Female#82 [67-101]	.4293338	.0079472	.4088628	.4498048
Female#184 [>101]	.2349625	.0078636	.2147069	.2552181

```
. ratio (One: n1Case/nLists) (OneOrTwo: n1OR2Cases/nLists), level(99) fvwrap(1) over(Gender PSI)
```

Ratio estimation   Number of obs = **94,005**

One: **n1Case/nLists**
OneOrTwo: **n1OR2Cases/nLists**

	Ratio	Linearized std. err.	[99% conf.	interval]
c.One@Gender#PSI				
Male#Cardiothoracic Surgery	.7337486	.0052035	.720345	.7471521
Male#Colorectal Surgery	.4839906	.006793	.4664926	.5014886
Male#Gastroenterology	.6850065	.0188982	.636327	.733686
Male#General Surgery	.4308025	.002454	.4244814	.4371236
Male#Gynecological Oncology	.32548	.0093561	.3013798	.3495802
Male#Neurosurgery	.4875114	.0048367	.4750527	.4999701
Male#OB/GYN	.6972957	.0028383	.6899847	.7046067
Male#OMFS	.7091742	.010993	.6808575	.7374909
Male#Ophthalmology	.1883696	.0023321	.1823624	.1943768
Male#Orthopedic Surgery	.3195927	.0019192	.3146491	.3245363
Male#Otolaryngology	.4241607	.0038665	.4142011	.4341202
Male#Plastic Surgery	.5421076	.0047806	.5297934	.5544218
Male#Podiatric Surgery	.6170023	.0039741	.6067655	.627239
Male#Surgical Oncology	.4653962	.0111869	.43658	.4942124
Male#Urology	.547868	.0031017	.5398783	.5558576
Male#Vascular Surgery	.5830002	.0058741	.5678693	.598131
Female#Cardiothoracic Surgery	.9248826	.0108534	.8969256	.9528397
Female#Colorectal Surgery	.4693167	.0147099	.4314257	.5072077
Female#Gastroenterology	.783737	.0177759	.7379484	.8295257
Female#General Surgery	.4512027	.0055681	.43686	.4655454
Female#Gynecological Oncology	.409925	.0140706	.3736808	.4461692
Female#Neurosurgery	.6483926	.016902	.6048551	.6919301
Female#OB/GYN	.7439909	.0027394	.7369346	.7510473
Female#OMFS	.7586957	.0212409	.7039817	.8134096
Female#Ophthalmology	.2202567	.0046744	.2082161	.2322973
Female#Orthopedic Surgery	.391386	.0097641	.366235	.4165371
Female#Otolaryngology	.4266901	.0107793	.3989238	.4544564
Female#Plastic Surgery	.5980523	.0097583	.5729159	.6231886
Female#Podiatric Surgery	.7214304	.0088189	.6987141	.7441468
Female#Surgical Oncology	.4671071	.0175788	.421826	.5123881
Female#Urology	.4460045	.0161889	.404304	.4877051
Female#Vascular Surgery	.6725352	.0147765	.6344728	.7105977
c.OneOrTwo@Gender#PSI				
Male#Cardiothoracic Surgery	.9416378	.0029955	.9339217	.949354
Male#Colorectal Surgery	.7404117	.0065745	.7234766	.7573468
Male#Gastroenterology	.8623207	.0162145	.8205541	.9040874
Male#General Surgery	.6790404	.0027355	.671994	.6860869
Male#Gynecological Oncology	.5731379	.0130696	.5394723	.6068035
Male#Neurosurgery	.7635794	.0048471	.7510938	.7760651
Male#OB/GYN	.8957851	.0021466	.8902557	.9013144
Male#OMFS	.9066838	.0096238	.8818942	.9314735
Male#Ophthalmology	.2929619	.0032306	.2846402	.3012837
Male#Orthopedic Surgery	.5320859	.0025114	.525617	.5385549
Male#Otolaryngology	.6545017	.0045583	.64276	.6662434
Male#Plastic Surgery	.7685352	.0047439	.7563155	.7807549
Male#Podiatric Surgery	.8389543	.0032421	.8306032	.8473055
Male#Surgical Oncology	.7312848	.0120316	.7002928	.7622767
Male#Urology	.7880927	.0029335	.7805363	.7956491
Male#Vascular Surgery	.8291516	.0053038	.8154895	.8428136
Female#Cardiothoracic Surgery	.9988263	.0011842	.9957758	1.001877
Female#Colorectal Surgery	.7200953	.0129267	.6867976	.7533929
Female#Gastroenterology	.9550173	.0081881	.9339257	.9761089
Female#General Surgery	.7055845	.0058403	.6905405	.7206285
Female#Gynecological Oncology	.6732089	.0153946	.6335542	.7128636
Female#Neurosurgery	.900846	.0109727	.8725817	.9291103

Female#OB/GYN	.9288598	.0017662	.9243102	.9334094
Female#OMFS	.9521739	.0112313	.9232435	.9811044
Female#Ophthalmology	.3533546	.006375	.3369333	.3697759
Female#Orthopedic Surgery	.5962029	.0118816	.5655973	.6268085
Female#Otolaryngology	.6260675	.0132698	.591886	.6602491
Female#Plastic Surgery	.8568939	.0081539	.8358905	.8778973
Female#Podiatric Surgery	.9073594	.0063942	.8908887	.9238301
Female#Surgical Oncology	.7486925	.0145321	.7112595	.7861255
Female#Urology	.6772662	.0188179	.6287934	.7257389
Female#Vascular Surgery	.9049296	.0093493	.8808469	.9290123

```
.
. by Gender, sort : summarize nCases, detail                                  // Explains results below
```

-> Gender = Male

Cases the surgeon performed during the quarter

	Percentiles	Smallest		
1%	0	0		
5%	3	0		
10%	5	0	Obs	75,382
25%	12	0	Sum of wgt.	75,382
50%	29		Mean	48.4927
		Largest	Std. dev.	67.63257
75%	61	1264		
90%	108	1341	Variance	4574.165
95%	151	1407	Skewness	5.575416
99%	316	1439	Kurtosis	58.56633

-> Gender = Female

Cases the surgeon performed during the quarter

	Percentiles	Smallest		
1%	0	0		
5%	2	0		
10%	3	0	Obs	18,623
25%	7	0	Sum of wgt.	18,623
50%	13		Mean	27.98013
		Largest	Std. dev.	49.76513
75%	30	886		
90%	65	974	Variance	2476.568
95%	98	981	Skewness	7.60427
99%	199	988	Kurtosis	95.55109

```
.         ranksum nCases, by(Gender) porder                                  // Interpret Wilcoxon-Mann-Whitney as AUC (WMWodds)
```

Two-sample Wilcoxon rank-sum (Mann-Whitney) test

Gender	Obs	Rank sum	Expected
Male	75382	3.761e+09	3.543e+09
Female	18623	6.572e+08	8.753e+08
Combined	94005	4.419e+09	4.419e+09

Unadjusted variance	1.100e+13
Adjustment for ties	-2.892e+09
Adjusted variance	1.099e+13

```
H0: nCases(Gender==Male) = nCases(Gender==Female)
         z = 65.790
Prob > |z| = 0.0000

P{nCases(Gender==Male) > nCases(Gender==Female)} = 0.655

.          roctab Gender nCases, binomial level(99)

                      ROC                       Binomial exact
          Obs       area     Std. err.      [99% conf. interval]
     ------------------------------------------------------------
       94,005     0.3446       0.0022        0.34062     0.34862

.                 display round(100*(1-0.3446),0.1) "%, exact binomial 99% CI " ///
>                             round(100*(1-0.34862),0.1) ", " round(100*(1-0.34062),0.1)
65.5%, exact binomial 99% CI 65.1, 65.9

.
.
. * Create 20 categories nCases to evaluate functional association with % lists with 1 case
. use "Stata temp", clear

. sort nCases

. xtile n20Cases = nCases, nq(20)

.         by n20Cases, sort : egen long Totaln1Case = total(n1Case)

.         by n20Cases, sort : egen long TotalLists  = total(nLists)

.         by n20Cases, sort : egen double Med20Cases = pctile(nCases), p(50)

. drop if n20Cases[_n] == n20Cases[_n+1]
(93,985 observations deleted)

. generate double Proportion = round( Totaln1Case / TotalLists, 0.01 )

. list n20Cases Totaln1Case TotalLists Med20Cases Proportion, nocompress abbreviate(11) noobs
```

n20Cases	Totaln1Case	TotalLists	Med20Cases	Proportion
1	11404	11882	2	.96
2	9397	10337	4	.91
3	21192	24311	5	.87
4	25380	30138	7	.84
5	27568	34219	9	.81
6	44031	56805	12	.78
7	29210	39163	14	.75
8	44253	61723	17	.72
9	41526	61184	20	.68
10	50035	77184	23	.65
11	45469	74989	27	.61
12	49486	87658	32	.56
13	48705	94989	37	.51
14	47933	102485	44	.47
15	43480	104664	51	.42
16	42758	120040	61	.36
17	36601	121771	73	.3
18	32020	133016	90	.24
19	23304	132783	117	.18
20	15627	129849	197	.12

```
. lowess Proportion Med20Cases, logit               // Nonlinear association with, without logit

. lowess Proportion Med20Cases

.
. *                 Reshape the 20 records to 40 rows, one for list has 1 case and one for list has >1 case
. *                 https://www.stata.com/support/faqs/statistics/logistic-regression-with-grouped-data
. generate w0  = TotalLists - Totaln1Case

. rename Totaln1Case w1

. generate id = _n

. reshape long w, i(id) j(y)
(j = 0 1)

Data                               Wide   ->   Long
-----------------------------------------------------------------------------

Number of observations               20   ->   40
Number of variables                  20   ->   20
j variable (2 values)                     ->   y
xij variables:
                                  w0 w1   ->   w
-----------------------------------------------------------------------------

.         label variable y "1 = List 1 case, 0 = otherwise"

. rename w FreqWeights

.         label variable FreqWeights "Count of lists, use with fw"

.
. boxtid logit y Med20Cases [fweight = FreqWeights]           // https://FDshort.com/30YXSCm

Iteration 0:  Deviance =   1752550
Iteration 1:  Deviance =   1752546 (change = -3.706807)
Iteration 2:  Deviance =   1752546 (change = -.0115773)
Iteration 3:  Deviance =   1752546 (change = -.0000342)
-> gen double IMed2__1 = X^0.1379-.8875242643 if e(sample)
-> gen double IMed2__2 = X^0.1379*ln(X)+.7678171618 if e(sample)
   (where: X = Med20Cases/100)

[Total iterations: 3]

Box-Tidwell regression model

Logistic regression                                    Number of obs = 1,509,190
                                                       LR chi2(2)    = 328348.79
                                                       Prob > chi2   =    0.0000
Log likelihood = -876273.06                            Pseudo R2     =    0.1578

------------------------------------------------------------------------------
           y | Coefficient  Std. err.      z    P>|z|     [95% conf. interval]
-------------+----------------------------------------------------------------
    IMed2__1 |  -9.758152    .231817   -42.09   0.000     -10.2125   -9.303799
    IMed2_p1 |  -.0000145   .0363672    -0.00   1.000     -.071293     .071264
       _cons |  -.1192652   .0022375   -53.30   0.000    -.1236507   -.1148798
------------------------------------------------------------------------------

------------------------------------------------------------------------------
Med20Cases   |  -.0219571   .0000512  -428.61 Nonlin. dev. 44204.491(P = 0.000)
          p1 |   .1379223   .0037255
------------------------------------------------------------------------------
Deviance: 1.75e+06.
```

```
.         display 1/0.1379233                                     // Transform 1/7.25 power; will instead use categories
7.2504066

.
.
. * Boxplot showing Male/Female without and with category of decile
. use "Stata temp", clear

.         sort PNPII FNI BinQtr                            // confirming expected structure

.         tabulate nDecCases Gender if nLists > 0, column  // males left-skewed mode 10th, females right-skewed mode 2nd
```

Key
frequency<br>*column percentage*

10 quantiles of nCases	0 Male 1 Female<br>Male	 Female	Total
2 [<5]	5,808<br>7.79	2,568<br>14.03	8,376<br>9.02
6 [5- 8]	6,558<br>8.80	3,308<br>18.08	9,866<br>10.63
11 [9-13]	7,308<br>9.80	3,266<br>17.85	10,574<br>11.39
16 [14-18]	6,584<br>8.83	2,067<br>11.29	8,651<br>9.32
22 [19-25]	7,713<br>10.35	1,749<br>9.56	9,462<br>10.19
30 [26-34]	7,772<br>10.42	1,271<br>6.94	9,043<br>9.74
41 [35-47]	8,075<br>10.83	1,174<br>6.41	9,249<br>9.96
56 [48-66]	7,980<br>10.70	1,102<br>6.02	9,082<br>9.78
82 [67-101]	8,291<br>11.12	921<br>5.03	9,212<br>9.92
184 [>101]	8,465<br>11.35	875<br>4.78	9,340<br>10.06
Total	74,554<br>100.00	18,301<br>100.00	92,855<br>100.00

```
.
. gen double Perc1CaseF = 100* n1Case / nLists if Gender == 1
(75,704 missing values generated)

. gen double Perc1CaseM = 100* n1Case / nLists if Gender == 0
(19,451 missing values generated)

.
. * https://statatexblog.com/2019/10/08/create-duplicate-observations-in-stata/
. expand 2, gen(dupindicator)
(94,005 observations created)

.         replace nDecCases = 0 if dupindicator == 1
(94,005 real changes made)

.         label define LabelnDecCasesRev 0 "Pooled"                                                     ///
>                                         1 "1: 2 [<5]"       2 "2: 6 [5-8]"    3 "3: 11 [9-13]"  4 "4: 16 [14-18]" ///
>                                                             5 "5: 22 [19-25]"   6 "6: 30 [26-34]" 7 "7: 41 [35-47]" 8 "8: 56 [48-66]" ///
>                                                             9 "9: 82 [67-101]" 10 "10: 184 [>101]"

. label values nDecCases LabelnDecCasesRev

.
. * https://www.stata.com/support/faqs/graphics/gph/graphdocs/box-plot-of-two-variables-by-categorical-variable/index.html
. graph hbox Perc1CaseM Perc1CaseF, over(nDecCases) nooutsides note("") legend(label(1 "Male") label(2 "Female"))  ///
>            l2title(Surgeons' Cases per Quarter) l1title(Decile: Median [Range])                              ///
>                    ytitle(Percentage of Lists with One Case) xsize(5) ysize(6) alsize(0)

.
.
. * Reshape to two rows per surgeon-quarter, following preceding Stata FAQ link
. use "Stata temp", clear

.
. generate w0  = nLists - n1Case

. rename n1Case w1

. generate id = _n

. reshape long w, i(id) j(y)
(j = 0 1)

Data                               Wide   ->   Long
-----------------------------------------------------------------------------
Number of observations           94,005   ->   188,010
Number of variables                  15   ->   15
j variable (2 values)                     ->   y
xij variables:
                                  w0 w1   ->   w
-----------------------------------------------------------------------------

.         label variable y "1 = List 1 case, 0 = otherwise"

. rename w FreqWeights
```

```
.         label variable FreqWeights "Count of lists, use with fw"

.
. * Listed in sequence of second column of Table 4, top to bottom
. logistic y i.Gender [fweight = FreqWeights], vce(robust) level(99)
```

Logistic regression — Number of obs = **1,509,190**; Wald chi2(**1**) = **8082.61**; Prob > chi2 = **0.0000**; Pseudo R2 = **0.0039**

Log pseudolikelihood = **-1036385.2**

y	Odds ratio	Robust std. err.	z	P>|z|	[99% conf.	interval]
Gender						
Female	**1.520201**	**.0070823**	**89.90**	**0.000**	**1.502067**	**1.538554**
_cons	**.7913699**	**.001402**	**-132.07**	**0.000**	**.7877667**	**.7949896**

Note: **_cons** estimates baseline odds.

```
.         margins   Gender, atmeans level(99)
```

Adjusted predictions — Number of obs = **1,509,190**
Model VCE: **Robust**

Expression: **Pr(y), predict()**
At: 0.Gender = **.8560175** (mean)
    1.Gender = **.1439825** (mean)

	Margin	Delta-method std. err.	z	P>|z|	[99% conf.	interval]
Gender						
Male	**.441768**	**.0004369**	**1011.12**	**0.000**	**.4406426**	**.4428934**
Female	**.5460821**	**.001068**	**511.29**	**0.000**	**.5433309**	**.5488332**

```
.         margins r.Gender, atmeans level(99)          // https://www.stata.com/manuals13/rmarginscontrast.pdf#page=4
```

Contrasts of adjusted predictions — Number of obs = **1,509,190**
Model VCE: **Robust**

Expression: **Pr(y), predict()**
At: 0.Gender = **.8560175** (mean)
    1.Gender = **.1439825** (mean)

	df	chi2	P>chi2
Gender	**1**	**8171.58**	**0.0000**

	Contrast	Delta-method std. err.	[99% conf.	interval]
Gender				
(Female vs Male)	**.104314**	**.001154**	**.1013416**	**.1072864**

```
.         display "Absolute risk difference 0.1043141 equals 0.5460821 - 0.441768"
Absolute risk difference 0.1043141 equals 0.5460821 - 0.441768

. logistic y i.Gender##i.nDecCases i.PSI i.BinQtr [fweight = FreqWeights], vce(cluster FNI) level(99)

Logistic regression                                     Number of obs = 1,509,190
                                                        Wald chi2(45) =  11769.96
                                                        Prob > chi2   =    0.0000
Log pseudolikelihood = -862891.02                       Pseudo R2     =    0.1707

                                              (Std. err. adjusted for 551 clusters in FNI)
```

y	Odds ratio	Robust std. err.	z	P>|z|	[99% conf. interval]	
**Gender**						
Female	.9964426	.0678053	-0.05	0.958	.8362378	1.187339
**nDecCases**						
6 [5- 8]	.4037783	.0162791	-22.49	0.000	.3639499	.4479652
11 [9-13]	.2486317	.0104129	-33.23	0.000	.223206	.2769538
16 [14-18]	.1781139	.0080067	-38.38	0.000	.1586393	.1999791
22 [19-25]	.1343323	.0062997	-42.81	0.000	.1190472	.1515799
30 [26-34]	.0982578	.0045991	-49.57	0.000	.0870977	.1108479
41 [35-47]	.0679497	.0032727	-55.83	0.000	.0600218	.0769248
56 [48-66]	.0450486	.0022039	-63.37	0.000	.0397148	.0510987
82 [67-101]	.0277139	.0014552	-68.29	0.000	.024208	.0317274
184 [>101]	.0154736	.0009089	-70.97	0.000	.013301	.018001
**Gender#nDecCases**						
Female# 6 [5- 8]	.9220059	.0644746	-1.16	0.246	.7700284	1.103979
Female#11 [9-13]	.9042685	.0656121	-1.39	0.165	.7501166	1.090099
Female#16 [14-18]	.9480553	.0685591	-0.74	0.461	.7869309	1.14217
Female#22 [19-25]	.8783223	.0697507	-1.63	0.102	.7158408	1.077684
Female#30 [26-34]	.8380161	.0636882	-2.33	0.020	.6890249	1.019224
Female#41 [35-47]	.8841512	.0695134	-1.57	0.117	.7220628	1.082625
Female#56 [48-66]	.8641578	.0685946	-1.84	0.066	.7043622	1.060206
Female#82 [67-101]	.8366175	.0745228	-2.00	0.045	.6650896	1.052383
Female#184 [>101]	1.181678	.1782281	1.11	0.268	.8012619	1.742704
**PSI**						
Colorectal Surgery	.6545697	.0586682	-4.73	0.000	.5196263	.8245569
Gastroenterology	.5430937	.098082	-3.38	0.001	.3410709	.8647784
General Surgery	.5552061	.0311409	-10.49	0.000	.4805176	.6415037
Gynecological Oncology	.4161793	.0428206	-8.52	0.000	.3192867	.5424755
Neurosurgery	.501034	.0328933	-10.53	0.000	.4230831	.593347
OB/GYN	.6171805	.0401052	-7.43	0.000	.5220591	.7296334
OMFS	.5532259	.0790341	-4.14	0.000	.3829032	.7993114
Ophthalmology	.2109142	.0235836	-13.92	0.000	.1581324	.2813136
Orthopedic Surgery	.3786773	.0215762	-17.04	0.000	.3269866	.4385393
Otolaryngology	.3693047	.0281488	-13.07	0.000	.303472	.4494187
Plastic Surgery	.620677	.0449121	-6.59	0.000	.5151326	.7478462
Podiatric Surgery	.3695453	.0245764	-14.97	0.000	.311366	.4385955
Surgical Oncology	.5301951	.0563141	-5.97	0.000	.40329	.697034
Urology	.4684739	.0305644	-11.62	0.000	.3960051	.5542044
Vascular Surgery	.5721718	.0488642	-6.54	0.000	.4591882	.7129551
**BinQtr**						
2	1.000366	.0106677	0.03	0.973	.9732622	1.028225
3	.9239527	.0107605	-6.79	0.000	.8966471	.9520899
4	.9651168	.0113395	-3.02	0.003	.9363458	.9947719
5	.9853352	.0113908	-1.28	0.201	.9564269	1.015117
6	.9749324	.0124212	-1.99	0.046	.9434568	1.007458
7	.9278186	.0123246	-5.64	0.000	.8966095	.960114
8	.9302241	.0121622	-5.53	0.000	.8994179	.9620855
9	.9301553	.0141762	-4.75	0.000	.8943474	.967397
10	.9392567	.0134182	-4.39	0.000	.9053218	.9744635
11	.8875901	.0134478	-7.87	0.000	.8536181	.922914

12	.8909934	.0147803	-6.96	0.000	.8537238	.92989
_cons	32.09953	2.219221	50.17	0.000	26.86327	38.35646

Note: **_cons** estimates baseline odds.

```
.         margins r.Gender, atmeans level(99)
```

Contrasts of adjusted predictions   Number of obs = **1,509,190**
Model VCE: **Robust**

Expression: **Pr(y), predict()**

```
At: 0.Gender     = .8560175 (mean)
    1.Gender     = .1439825 (mean)
    1.nDecCases  = .0147225 (mean)
    2.nDecCases  = .0360783 (mean)
    3.nDecCases  = .0603131 (mean)
    4.nDecCases  = .0668478 (mean)
    5.nDecCases  = .0916836 (mean)
    6.nDecCases  = .1077711 (mean)
    7.nDecCases  = .1308477 (mean)
    8.nDecCases  = .1488905 (mean)
    9.nDecCases  = .1688237 (mean)
    10.nDecCases = .1740218 (mean)
    1.PSI        = .0292886 (mean)
    2.PSI        = .0207727 (mean)
    3.PSI        = .0029241 (mean)
    4.PSI        = .1712415 (mean)
    5.PSI        = .0112961 (mean)
    6.PSI        = .0476666 (mean)
    7.PSI        = .1273372 (mean)
    8.PSI        = .0049344 (mean)
    9.PSI        =  .109189 (mean)
    10.PSI       = .2258967 (mean)
    11.PSI       = .0586487 (mean)
    12.PSI       = .0494782 (mean)
    13.PSI       = .0370006 (mean)
    14.PSI       = .0096741 (mean)
    15.PSI       = .0717219 (mean)
    16.PSI       = .0229295 (mean)
    1.BinQtr     = .0833765 (mean)
    2.BinQtr     = .0846182 (mean)
    3.BinQtr     = .0776483 (mean)
    4.BinQtr     = .0843916 (mean)
    5.BinQtr     = .0846176 (mean)
    6.BinQtr     = .0854021 (mean)
    7.BinQtr     =   .08205 (mean)
    8.BinQtr     = .0846719 (mean)
    9.BinQtr     = .0835793 (mean)
    10.BinQtr    = .0859521 (mean)
    11.BinQtr    = .0821136 (mean)
    12.BinQtr    = .0815789 (mean)
```

	df	chi2	P>chi2
Gender	1	4.71	0.0299

	Contrast	Delta-method std. err.	[99% conf.	interval]
Gender (Female vs Male)	-.0211863	.0097585	-.0463227	.00395

```
. logistic y i.Gender##i.nDecCases i.PSI i.BinQtr [fweight = FreqWeights], vce(robust)      level(99)

Logistic regression                                     Number of obs = 1,509,190
                                                        Wald chi2(45) = 263248.70
                                                        Prob > chi2   =    0.0000
Log pseudolikelihood = -862891.02                       Pseudo R2     =    0.1707
```

y	Odds ratio	Robust std. err.	z	P>|z|	[99% conf.	interval]
Gender						
Female	.9964426	.0604583	-0.06	0.953	.8522717	1.165001
nDecCases						
6 [5- 8]	.4037783	.0145519	-25.16	0.000	.3679822	.4430565
11 [9-13]	.2486317	.0085129	-40.65	0.000	.2276431	.2715555
16 [14-18]	.1781139	.006027	-50.99	0.000	.1632466	.1943351
22 [19-25]	.1343323	.0044955	-59.99	0.000	.1232378	.1464256
30 [26-34]	.0982578	.0032753	-69.60	0.000	.0901732	.1070672
41 [35-47]	.0679497	.0022601	-80.84	0.000	.0623705	.0740281
56 [48-66]	.0450486	.0014983	-93.21	0.000	.0413498	.0490781
82 [67-101]	.0277139	.000923	-107.67	0.000	.0254355	.0301963
184 [>101]	.0154736	.0005182	-124.48	0.000	.0141948	.0168675
Gender#nDecCases						
Female# 6 [5- 8]	.9220059	.0608439	-1.23	0.218	.7778787	1.092837
Female#11 [9-13]	.9042685	.0571112	-1.59	0.111	.7685025	1.064019
Female#16 [14-18]	.9480553	.0596416	-0.85	0.396	.8062299	1.11483
Female#22 [19-25]	.8783223	.0548305	-2.08	0.038	.7478585	1.031545
Female#30 [26-34]	.8380161	.0523268	-2.83	0.005	.713512	.9842454
Female#41 [35-47]	.8841512	.0550663	-1.98	0.048	.7531025	1.038004
Female#56 [48-66]	.8641578	.0538247	-2.34	0.019	.7360648	1.014542
Female#82 [67-101]	.8366175	.0526876	-2.83	0.005	.711339	.9839596
Female#184 [>101]	1.181678	.0758434	2.60	0.009	1.001613	1.394114
PSI						
Colorectal Surgery	.6545697	.010672	-25.99	0.000	.6276496	.6826444
Gastroenterology	.5430937	.0203164	-16.32	0.000	.4932044	.5980295
General Surgery	.5552061	.0066182	-49.36	0.000	.5384178	.5725178
Gynecological Oncology	.4161793	.0084359	-43.25	0.000	.3950074	.4384859
Neurosurgery	.501034	.0067639	-51.19	0.000	.4839107	.5187633
OB/GYN	.6171805	.0076747	-38.81	0.000	.5977251	.6372691
OMFS	.5532259	.0164604	-19.90	0.000	.5124107	.5972921
Ophthalmology	.2109142	.0027682	-118.58	0.000	.2039029	.2181666
Orthopedic Surgery	.3786773	.0044631	-82.39	0.000	.3673537	.3903498
Otolaryngology	.3693047	.0049112	-74.91	0.000	.3568685	.3821744
Plastic Surgery	.620677	.0084609	-34.99	0.000	.5992612	.6428581
Podiatric Surgery	.3695453	.005376	-68.43	0.000	.3559538	.3836558
Surgical Oncology	.5301951	.010907	-30.84	0.000	.5028319	.5590474
Urology	.4684739	.005966	-59.54	0.000	.4533558	.4840962
Vascular Surgery	.5721718	.0091196	-35.03	0.000	.549157	.5961511
BinQtr						
2	1.000366	.0089669	0.04	0.967	.9775337	1.023732
3	.9239527	.0084928	-8.60	0.000	.9023336	.9460899
4	.9651168	.0086841	-3.95	0.000	.9430052	.9877469
5	.9853352	.0088595	-1.64	0.100	.9627768	1.008422
6	.9749324	.0087499	-2.83	0.005	.9526527	.9977331
7	.9278186	.0084055	-8.27	0.000	.906418	.9497244
8	.9302241	.0083664	-8.04	0.000	.9089215	.952026
9	.9301553	.0083918	-8.03	0.000	.9087888	.9520242
10	.9392567	.008398	-7.01	0.000	.9178721	.9611394
11	.8875901	.0080201	-13.20	0.000	.8671703	.9084906
12	.8909934	.0080897	-12.71	0.000	.8703975	.9120766
_cons	32.09953	1.128967	98.63	0.000	29.31934	35.14335

```
Note: _cons estimates baseline odds.

.         margins r.Gender, atmeans level(99)

Contrasts of adjusted predictions                    Number of obs = 1,509,190
Model VCE: Robust

Expression: Pr(y), predict()
At: 0.Gender     = .8560175 (mean)
    1.Gender     = .1439825 (mean)
    1.nDecCases  = .0147225 (mean)
    2.nDecCases  = .0360783 (mean)
    3.nDecCases  = .0603131 (mean)
    4.nDecCases  = .0668478 (mean)
    5.nDecCases  = .0916836 (mean)
    6.nDecCases  = .1077711 (mean)
    7.nDecCases  = .1308477 (mean)
    8.nDecCases  = .1488905 (mean)
    9.nDecCases  = .1688237 (mean)
    10.nDecCases = .1740218 (mean)
    1.PSI        = .0292886 (mean)
    2.PSI        = .0207727 (mean)
    3.PSI        = .0029241 (mean)
    4.PSI        = .1712415 (mean)
    5.PSI        = .0112961 (mean)
    6.PSI        = .0476666 (mean)
    7.PSI        = .1273372 (mean)
    8.PSI        = .0049344 (mean)
    9.PSI        =  .109189 (mean)
    10.PSI       = .2258967 (mean)
    11.PSI       = .0586487 (mean)
    12.PSI       = .0494782 (mean)
    13.PSI       = .0370006 (mean)
    14.PSI       = .0096741 (mean)
    15.PSI       = .0717219 (mean)
    16.PSI       = .0229295 (mean)
    1.BinQtr     = .0833765 (mean)
    2.BinQtr     = .0846182 (mean)
    3.BinQtr     = .0776483 (mean)
    4.BinQtr     = .0843916 (mean)
    5.BinQtr     = .0846176 (mean)
    6.BinQtr     = .0854021 (mean)
    7.BinQtr     =   .08205 (mean)
    8.BinQtr     = .0846719 (mean)
    9.BinQtr     = .0835793 (mean)
    10.BinQtr    = .0859521 (mean)
    11.BinQtr    = .0821136 (mean)
    12.BinQtr    = .0815789 (mean)
```

	df	chi2	P>chi2
Gender	1	188.71	0.0000

	Contrast	Delta-method std. err.	[99% conf.	interval]
Gender (Female vs Male)	-.0211863	.0015423	-.0251589	-.0172137

```
. logistic y i.Gender  i.nDecCases i.PSI i.BinQtr [fweight = FreqWeights], vce(cluster FNI) level(99)
```

Logistic regression   Number of obs = 1,509,190
                                                   Wald chi2(36) = 10706.43
                                                   Prob > chi2   =   0.0000
Log pseudolikelihood = -863006.6   Pseudo R2   =   0.1705

(Std. err. adjusted for 551 clusters in FNI)

y	Odds ratio	Robust std. err.	z	P>|z|	[99% conf.	interval]
Gender						
Female	.8933827	.0252044	-4.00	0.000	.8307633	.960722
nDecCases						
6 [5- 8]	.3942594	.0141338	-25.96	0.000	.3594835	.4323993
11 [9-13]	.2411342	.0085389	-40.17	0.000	.2201129	.2641632
16 [14-18]	.1746211	.0070088	-43.48	0.000	.1574696	.1936407
22 [19-25]	.1294469	.0055232	-47.92	0.000	.115974	.144485
30 [26-34]	.0941905	.0040097	-55.49	0.000	.0844083	.1051065
41 [35-47]	.0656479	.0028819	-62.04	0.000	.0586289	.0735073
56 [48-66]	.0434379	.001952	-69.80	0.000	.0386901	.0487685
82 [67-101]	.0266946	.0012954	-74.67	0.000	.0235581	.0302487
184 [>101]	.0152721	.000909	-70.25	0.000	.0131013	.0178027
PSI						
Colorectal Surgery	.6539128	.0591476	-4.70	0.000	.5180053	.8254779
Gastroenterology	.5393038	.0969528	-3.43	0.001	.339411	.8569215
General Surgery	.55216	.0310437	-10.56	0.000	.4777173	.6382032
Gynecological Oncology	.4117177	.042216	-8.65	0.000	.3161517	.5361713
Neurosurgery	.4998491	.0328618	-10.55	0.000	.4219819	.592085
OB/GYN	.6159265	.040181	-7.43	0.000	.5206557	.7286301
OMFS	.5508273	.0789809	-4.16	0.000	.3807273	.7969238
Ophthalmology	.2111669	.0239531	-13.71	0.000	.1576642	.2828256
Orthopedic Surgery	.3775172	.0216152	-17.01	0.000	.3257512	.4375095
Otolaryngology	.369344	.028109	-13.09	0.000	.3035949	.4493322
Plastic Surgery	.6172617	.0447016	-6.66	0.000	.5122197	.743845
Podiatric Surgery	.3687485	.0245204	-15.00	0.000	.3107012	.4376405
Surgical Oncology	.5256089	.0559947	-6.04	0.000	.3994732	.6915727
Urology	.468514	.0305793	-11.62	0.000	.3960122	.5542894
Vascular Surgery	.5709513	.0488131	-6.56	0.000	.4580989	.7116049
BinQtr						
2	1.000803	.0107051	0.08	0.940	.9736046	1.028761
3	.9243092	.0107542	-6.76	0.000	.8970191	.9524296
4	.9655017	.0113216	-2.99	0.003	.9367752	.9951091
5	.9854763	.0113336	-1.27	0.203	.9567111	1.015106
6	.9749018	.0124506	-1.99	0.047	.9433529	1.007506
7	.9280076	.0123135	-5.63	0.000	.8968259	.9602734
8	.9300822	.012136	-5.55	0.000	.8993414	.9618737
9	.930075	.0141641	-4.76	0.000	.8942971	.9672844
10	.9392109	.0133724	-4.40	0.000	.9053899	.9742952
11	.8878062	.013462	-7.85	0.000	.8537988	.9231681
12	.8910776	.0148024	-6.94	0.000	.8537535	.9300334
_cons	33.25268	2.162238	53.89	0.000	28.12457	39.31583

Note: **_cons** estimates baseline odds.

```
.         margins r.Gender, atmeans level(99)

Contrasts of adjusted predictions                    Number of obs = 1,509,190
Model VCE: Robust

Expression: Pr(y), predict()
At: 0.Gender     = .8560175 (mean)
    1.Gender     = .1439825 (mean)
    1.nDecCases  = .0147225 (mean)
    2.nDecCases  = .0360783 (mean)
    3.nDecCases  = .0603131 (mean)
    4.nDecCases  = .0668478 (mean)
    5.nDecCases  = .0916836 (mean)
    6.nDecCases  = .1077711 (mean)
    7.nDecCases  = .1308477 (mean)
    8.nDecCases  = .1488905 (mean)
    9.nDecCases  = .1688237 (mean)
    10.nDecCases = .1740218 (mean)
    1.PSI        = .0292886 (mean)
    2.PSI        = .0207727 (mean)
    3.PSI        = .0029241 (mean)
    4.PSI        = .1712415 (mean)
    5.PSI        = .0112961 (mean)
    6.PSI        = .0476666 (mean)
    7.PSI        = .1273372 (mean)
    8.PSI        = .0049344 (mean)
    9.PSI        =  .109189 (mean)
    10.PSI       = .2258967 (mean)
    11.PSI       = .0586487 (mean)
    12.PSI       = .0494782 (mean)
    13.PSI       = .0370006 (mean)
    14.PSI       = .0096741 (mean)
    15.PSI       = .0717219 (mean)
    16.PSI       = .0229295 (mean)
    1.BinQtr     = .0833765 (mean)
    2.BinQtr     = .0846182 (mean)
    3.BinQtr     = .0776483 (mean)
    4.BinQtr     = .0843916 (mean)
    5.BinQtr     = .0846176 (mean)
    6.BinQtr     = .0854021 (mean)
    7.BinQtr     =   .08205 (mean)
    8.BinQtr     = .0846719 (mean)
    9.BinQtr     = .0835793 (mean)
    10.BinQtr    = .0859521 (mean)
    11.BinQtr    = .0821136 (mean)
    12.BinQtr    = .0815789 (mean)
```

	df	chi2	P>chi2
Gender	1	16.29	0.0001

	Contrast	Delta-method std. err.	[99% conf.	interval]
Gender (Female vs Male)	-.027739	.0068722	-.0454407	-.0100373

```
. logistic y i.Gender  i.nDecCases i.PSI          [fweight = FreqWeights], vce(cluster FNI) level(99)

Logistic regression                                   Number of obs = 1,509,190
                                                      Wald chi2(25) =   9530.20
                                                      Prob > chi2   =    0.0000
Log pseudolikelihood = -863230.75                     Pseudo R2     =    0.1703

                                             (Std. err. adjusted for 551 clusters in FNI)
```

y	Odds ratio	Robust std. err.	z	P>|z|	[99% conf.	interval]
**Gender**						
Female	.8919547	.0251295	-4.06	0.000	.8295183	.9590906
**nDecCases**						
6 [5- 8]	.3939142	.0140946	-26.04	0.000	.3592316	.4319452
11 [9-13]	.2409102	.0085173	-40.26	0.000	.2199404	.2638794
16 [14-18]	.1744859	.0069919	-43.57	0.000	.1573743	.1934581
22 [19-25]	.1293333	.0054974	-48.12	0.000	.1159205	.144298
30 [26-34]	.0941988	.003991	-55.76	0.000	.0844599	.1050608
41 [35-47]	.0656635	.0028695	-62.32	0.000	.0586731	.0734868
56 [48-66]	.0434635	.0019467	-70.01	0.000	.0387276	.0487787
82 [67-101]	.0267068	.0012884	-75.10	0.000	.023586	.0302404
184 [>101]	.0153041	.000907	-70.53	0.000	.0131375	.017828
**PSI**						
Colorectal Surgery	.6539899	.059104	-4.70	0.000	.5181697	.8254107
Gastroenterology	.53991	.0968019	-3.44	0.001	.3402141	.8568215
General Surgery	.5524123	.0311191	-10.53	0.000	.4777991	.6386772
Gynecological Oncology	.4122367	.0422117	-8.65	0.000	.3166639	.5366544
Neurosurgery	.5001071	.0329	-10.53	0.000	.4221536	.5924553
OB/GYN	.6167698	.0402885	-7.40	0.000	.5212543	.7297876
OMFS	.5511302	.0787654	-4.17	0.000	.381398	.7963977
Ophthalmology	.2113052	.0239863	-13.69	0.000	.1577337	.2830713
Orthopedic Surgery	.3776808	.0216589	-16.98	0.000	.3258161	.4378015
Otolaryngology	.369679	.0281864	-13.05	0.000	.3037604	.4499025
Plastic Surgery	.6174341	.044756	-6.65	0.000	.5122731	.7441828
Podiatric Surgery	.3693334	.0245786	-14.97	0.000	.3111522	.4383938
Surgical Oncology	.524665	.05581	-6.06	0.000	.3989204	.6900457
Urology	.4688372	.0306279	-11.60	0.000	.3962255	.5547555
Vascular Surgery	.5712052	.0488603	-6.55	0.000	.45825	.7120032
_cons	31.44233	2.019645	53.68	0.000	26.64765	37.09971

```
Note: _cons estimates baseline odds.

.         margins r.Gender, atmeans level(99)

Contrasts of adjusted predictions                     Number of obs = 1,509,190
Model VCE: Robust

Expression: Pr(y), predict()
At: 0.Gender     = .8560175 (mean)
    1.Gender     = .1439825 (mean)
    1.nDecCases  = .0147225 (mean)
    2.nDecCases  = .0360783 (mean)
    3.nDecCases  = .0603131 (mean)
    4.nDecCases  = .0668478 (mean)
    5.nDecCases  = .0916836 (mean)
    6.nDecCases  = .1077711 (mean)
    7.nDecCases  = .1308477 (mean)
    8.nDecCases  = .1488905 (mean)
    9.nDecCases  = .1688237 (mean)
    10.nDecCases = .1740218 (mean)
    1.PSI        = .0292886 (mean)
    2.PSI        = .0207727 (mean)
    3.PSI        = .0029241 (mean)
```

```
    4.PSI        = .1712415 (mean)
    5.PSI        = .0112961 (mean)
    6.PSI        = .0476666 (mean)
    7.PSI        = .1273372 (mean)
    8.PSI        = .0049344 (mean)
    9.PSI        =  .109189 (mean)
    10.PSI       = .2258967 (mean)
    11.PSI       = .0586487 (mean)
    12.PSI       = .0494782 (mean)
    13.PSI       = .0370006 (mean)
    14.PSI       = .0096741 (mean)
    15.PSI       = .0717219 (mean)
    16.PSI       = .0229295 (mean)
```

	df	chi2	P>chi2
Gender	1	16.81	0.0000

	Contrast	Delta-method std. err.	[99% conf. interval]	
Gender (Female vs Male)	-.0281306	.0068612	-.0458038	-.0104575

```
. logistic y i.Gender  i.nDecCases                [fweight = FreqWeights], vce(cluster FNI) level(99)

Logistic regression                                  Number of obs = 1,509,190
                                                     Wald chi2(10) =   7627.10
                                                     Prob > chi2   =    0.0000
Log pseudolikelihood = -877934.57                    Pseudo R2     =    0.1562

                                       (Std. err. adjusted for 551 clusters in FNI)
```

y	Odds ratio	Robust std. err.	z	P>|z|	[99% conf. interval]	
Gender						
Female	.9117746	.0230801	-3.65	0.000	.8542209	.973206
nDecCases						
6 [5- 8]	.4039768	.0152131	-24.07	0.000	.3666309	.4451267
11 [9-13]	.2511852	.0090409	-38.38	0.000	.2289443	.2755868
16 [14-18]	.1813778	.0073419	-42.17	0.000	.1634189	.2013104
22 [19-25]	.1316929	.0055745	-47.89	0.000	.1180892	.1468638
30 [26-34]	.0940195	.0040079	-55.46	0.000	.0842424	.1049313
41 [35-47]	.0641482	.0028731	-61.32	0.000	.0571586	.0719926
56 [48-66]	.0416557	.0018965	-69.81	0.000	.0370463	.0468387
82 [67-101]	.0245947	.0012119	-75.19	0.000	.021663	.0279232
184 [>101]	.0116023	.0007043	-73.41	0.000	.0099228	.013566
_cons	15.10725	.6310698	65.00	0.000	13.56612	16.82346

Note: **_cons** estimates baseline odds.

```
.         margins r.Gender, atmeans level(99)

Contrasts of adjusted predictions                   Number of obs = 1,509,190
Model VCE: Robust

Expression: Pr(y), predict()
At: 0.Gender     = .8560175 (mean)
    1.Gender     = .1439825 (mean)
    1.nDecCases  = .0147225 (mean)
    2.nDecCases  = .0360783 (mean)
    3.nDecCases  = .0603131 (mean)
    4.nDecCases  = .0668478 (mean)
    5.nDecCases  = .0916836 (mean)
    6.nDecCases  = .1077711 (mean)
    7.nDecCases  = .1308477 (mean)
    8.nDecCases  = .1488905 (mean)
    9.nDecCases  = .1688237 (mean)
    10.nDecCases = .1740218 (mean)
```

	df	chi2	P>chi2
Gender	1	13.46	0.0002

	Contrast	Delta-method std. err.	[99% conf.	interval]
Gender (Female vs Male)	-.0227696	.0062066	-.0387568	-.0067823

```
. logistic y i.Gender  i.nDecCases                [fweight = FreqWeights], vce(robust)      level(99)

Logistic regression                                 Number of obs = 1,509,190
                                                    Wald chi2(10) = 250948.42
                                                    Prob > chi2   =    0.0000
Log pseudolikelihood = -877934.57                   Pseudo R2     =    0.1562
```

y	Odds ratio	Robust std. err.	z	P>|z|	[99% conf.	interval]
Gender						
Female	.9117746	.0048566	-17.34	0.000	.8993502	.9243706
nDecCases						
6 [5- 8]	.4039768	.0121344	-30.18	0.000	.3738992	.4364739
11 [9-13]	.2511852	.0071894	-48.27	0.000	.2333327	.2704037
16 [14-18]	.1813778	.0051433	-60.20	0.000	.1686017	.1951221
22 [19-25]	.1316929	.0036941	-72.27	0.000	.1225132	.1415605
30 [26-34]	.0940195	.0026265	-84.63	0.000	.0874916	.1010344
41 [35-47]	.0641482	.0017865	-98.62	0.000	.0597077	.068919
56 [48-66]	.0416557	.0011591	-114.22	0.000	.0387746	.0447509
82 [67-101]	.0245947	.000685	-133.03	0.000	.0228921	.026424
184 [>101]	.0116023	.0003253	-158.94	0.000	.0107939	.0124713
_cons	15.10725	.4157604	98.66	0.000	14.0734	16.21705

Note: **_cons** estimates baseline odds.

```
.         margins r.Gender, atmeans level(99)

Contrasts of adjusted predictions                   Number of obs = 1,509,190
Model VCE: Robust

Expression: Pr(y), predict()
At: 0.Gender     = .8560175 (mean)
    1.Gender     = .1439825 (mean)
    1.nDecCases  = .0147225 (mean)
    2.nDecCases  = .0360783 (mean)
    3.nDecCases  = .0603131 (mean)
    4.nDecCases  = .0668478 (mean)
    5.nDecCases  = .0916836 (mean)
    6.nDecCases  = .1077711 (mean)
    7.nDecCases  = .1308477 (mean)
    8.nDecCases  = .1488905 (mean)
    9.nDecCases  = .1688237 (mean)
    10.nDecCases = .1740218 (mean)
```

	df	chi2	P>chi2
Gender	1	303.18	0.0000

	Contrast	Delta-method std. err.	[99% conf.	interval]
Gender (Female vs Male)	-.0227696	.0013077	-.0261379	-.0194012

```
. logistic y i.Gender              i.PSI          [fweight = FreqWeights], vce(cluster FNI) level(99)

Logistic regression                                 Number of obs = 1,509,190
                                                    Wald chi2(16) =   1820.28
                                                    Prob > chi2   =    0.0000
Log pseudolikelihood = -958217.85                   Pseudo R2     =    0.0790

                                             (Std. err. adjusted for 551 clusters in FNI)
```

y	Odds ratio	Robust std. err.	z	P>|z|	[99% conf.	interval]
Gender						
Female	1.199163	.0407379	5.35	0.000	1.098689	1.308825
PSI						
Colorectal Surgery	.3213331	.0423273	-8.62	0.000	.2288759	.4511396
Gastroenterology	.8066671	.2489453	-0.70	0.486	.3643027	1.786184
General Surgery	.2666923	.0255337	-13.80	0.000	.2084043	.3412827
Gynecological Oncology	.1849538	.0279232	-11.18	0.000	.1253643	.2728681
Neurosurgery	.3463559	.0385015	-9.54	0.000	.2601175	.4611855
OB/GYN	.8394681	.0930508	-1.58	0.114	.6309654	1.116871
OMFS	.8747079	.1446105	-0.81	0.418	.5713732	1.339079
Ophthalmology	.083155	.0112583	-18.37	0.000	.0586722	.1178539
Orthopedic Surgery	.1688514	.0165798	-18.11	0.000	.1311175	.2174447
Otolaryngology	.2567794	.0313206	-11.15	0.000	.1875473	.3515683
Plastic Surgery	.4254931	.0462499	-7.86	0.000	.3215855	.5629742
Podiatric Surgery	.5972081	.0660646	-4.66	0.000	.449134	.7941005
Surgical Oncology	.2977201	.0464743	-7.76	0.000	.1991514	.4450749
Urology	.4216049	.0440447	-8.27	0.000	.3221361	.5517877
Vascular Surgery	.5073443	.0625761	-5.50	0.000	.3692544	.6970757
_cons	2.799153	.2691258	10.71	0.000	2.185102	3.585762

Note: _cons estimates baseline odds.

```
.         margins r.Gender, atmeans level(99)

Contrasts of adjusted predictions                    Number of obs = 1,509,190
Model VCE: Robust

Expression: Pr(y), predict()
At: 0.Gender = .8560175 (mean)
    1.Gender = .1439825 (mean)
    1.PSI    = .0292886 (mean)
    2.PSI    = .0207727 (mean)
    3.PSI    = .0029241 (mean)
    4.PSI    = .1712415 (mean)
    5.PSI    = .0112961 (mean)
    6.PSI    = .0476666 (mean)
    7.PSI    = .1273372 (mean)
    8.PSI    = .0049344 (mean)
    9.PSI    =  .109189 (mean)
    10.PSI   = .2258967 (mean)
    11.PSI   = .0586487 (mean)
    12.PSI   = .0494782 (mean)
    13.PSI   = .0370006 (mean)
    14.PSI   = .0096741 (mean)
    15.PSI   = .0717219 (mean)
    16.PSI   = .0229295 (mean)
```

	df	chi2	P>chi2
Gender	1	28.39	0.0000

	Contrast	Delta-method std. err.	[99% conf.	interval]
Gender (Female vs Male)	.0451936	.0084814	.023347	.0670402

```
.
.
. * Sensitivity analysis using 1 or 2 cases instead of 1 case
. use "Stata temp", clear

.
. generate w0  = nLists - n1OR2Cases

. rename n1OR2Cases w1

. generate id = _n

. reshape long w, i(id) j(y)
(j = 0 1)
```

Data	Wide	->	Long
Number of observations	94,005	->	188,010
Number of variables	15	->	15
j variable (2 values)		->	y
xij variables:			
	w0 w1	->	w

```
.         label variable y "1 = List 1 or 2 cases, 0 = otherwise"

. rename w FreqWeights

.         label variable FreqWeights "Count of lists, use with fw"

.
. * Listed in sequence of third column of Table 4, top to bottom
. logistic y i.Gender [fweight = FreqWeights], vce(robust) level(99)

Logistic regression                                     Number of obs = 1,509,190
                                                        Wald chi2(1)  =   6865.00
                                                        Prob > chi2   =    0.0000
Log pseudolikelihood = -952620.4                        Pseudo R2     =    0.0038

------------------------------------------------------------------------------
             |               Robust
           y | Odds ratio   std. err.      z    P>|z|     [99% conf. interval]
-------------+----------------------------------------------------------------
      Gender |
      Female |   1.548891   .0081793    82.86   0.000     1.527965    1.570104
       _cons |   1.921711   .0035634   352.27   0.000     1.912554    1.930912
------------------------------------------------------------------------------
Note: _cons estimates baseline odds.

.         margins r.Gender, atmeans level(99)

Contrasts of adjusted predictions                       Number of obs = 1,509,190
Model VCE: Robust

Expression: Pr(y), predict()
At: 0.Gender = .8560175 (mean)
    1.Gender = .1439825 (mean)

------------------------------------------------
             |         df        chi2     P>chi2
-------------+----------------------------------
      Gender |          1     7921.70     0.0000
------------------------------------------------

--------------------------------------------------------------------
                  |            Delta-method
                  |   Contrast   std. err.     [99% conf. interval]
------------------+-------------------------------------------------
           Gender |
(Female vs Male)  |   .0907891   .0010201      .0881616    .0934166
--------------------------------------------------------------------

. logistic y i.Gender##i.nDecCases i.PSI i.BinQtr [fweight = FreqWeights], vce(cluster FNI) level(99)

Logistic regression                                     Number of obs = 1,509,190
                                                        Wald chi2(45) =   9687.41
                                                        Prob > chi2   =    0.0000
Log pseudolikelihood = -712513.8                        Pseudo R2     =    0.2549

                                                (Std. err. adjusted for 551 clusters in FNI)
-------------------------------------------------------------------------------------------
                          |               Robust
                        y | Odds ratio   std. err.      z    P>|z|     [99% conf. interval]
--------------------------+----------------------------------------------------------------
                   Gender |
                   Female |     1.1576   .2950145     0.57   0.566     .6004345     2.23178
                          |
                nDecCases |
               6 [ 5- 8]  |   .2043552   .0270912   -11.98   0.000     .1452395    .2875322
              11 [ 9-13]  |   .0952839   .0129607   -17.28   0.000     .0671206    .1352643
              16 [14-18]  |   .0574817   .0080115   -20.49   0.000     .0401435    .0823085
              22 [19-25]  |   .0383681   .0054316   -23.03   0.000     .0266444    .0552503
```

30 [26-34]	.025859	.003663	-25.80	0.000	.0179536	.0372454
41 [35-47]	.016094	.0022909	-29.01	0.000	.0111538	.0232222
56 [48-66]	.0092468	.001322	-32.76	0.000	.0063982	.0133637
82 [67-101]	.0048144	.0006962	-36.90	0.000	.0033172	.0069874
184 [>101]	.0021357	.0003163	-41.52	0.000	.0014583	.0031276
Gender#nDecCases						
Female# 6 [5- 8]	.9359747	.234642	-0.26	0.792	.4907068	1.785279
Female#11 [9-13]	.8496172	.2123229	-0.65	0.514	.4463375	1.617273
Female#16 [14-18]	.8764163	.2260139	-0.51	0.609	.4510488	1.702932
Female#22 [19-25]	.7761227	.2057895	-0.96	0.339	.3920258	1.536548
Female#30 [26-34]	.6968821	.180895	-1.39	0.164	.3570902	1.360006
Female#41 [35-47]	.6871786	.1803299	-1.43	0.153	.3495488	1.350925
Female#56 [48-66]	.698638	.1827641	-1.37	0.170	.3561294	1.370555
Female#82 [67-101]	.7111783	.1938213	-1.25	0.211	.3524533	1.435011
Female#184 [>101]	1.025812	.3217407	0.08	0.935	.4573021	2.301084
PSI						
Colorectal Surgery	.3912282	.0566166	-6.48	0.000	.2694895	.5679609
Gastroenterology	.2887331	.1083471	-3.31	0.001	.1098288	.7590612
General Surgery	.3366293	.0365719	-10.02	0.000	.2544592	.4453338
Gynecological Oncology	.2480669	.0390986	-8.84	0.000	.165292	.3722939
Neurosurgery	.3531553	.0428747	-8.57	0.000	.2583177	.4828112
OB/GYN	.3585096	.0399733	-9.20	0.000	.2690116	.4777828
OMFS	.3512323	.0883448	-4.16	0.000	.183746	.671384
Ophthalmology	.0799592	.0123145	-16.40	0.000	.0537752	.1188927
Orthopedic Surgery	.1990651	.0204749	-15.69	0.000	.1527335	.2594513
Otolaryngology	.1812853	.023349	-13.26	0.000	.1301012	.2526061
Plastic Surgery	.3667087	.0473053	-7.78	0.000	.2630349	.5112451
Podiatric Surgery	.1747502	.0207736	-14.67	0.000	.1286575	.237356
Surgical Oncology	.3300266	.0500055	-7.32	0.000	.2233822	.4875838
Urology	.244235	.0246404	-13.97	0.000	.1883422	.3167147
Vascular Surgery	.3610069	.0560051	-6.57	0.000	.2420862	.5383454
BinQtr						
2	1.013511	.0118856	1.14	0.252	.9833529	1.044593
3	.9111873	.0147098	-5.76	0.000	.8740743	.9498762
4	.9558744	.0138482	-3.12	0.002	.9208612	.9922189
5	.9898352	.0147659	-0.68	0.493	.9525223	1.02861
6	.9914834	.0158163	-0.54	0.592	.951569	1.033072
7	.9227055	.0160113	-4.64	0.000	.8823711	.9648835
8	.9235837	.0164178	-4.47	0.000	.8822477	.9668564
9	.9367327	.0182519	-3.35	0.001	.8908791	.9849463
10	.9520286	.0174939	-2.68	0.007	.9080172	.9981733
11	.8871153	.0175758	-6.05	0.000	.8429788	.9335626
12	.8822919	.0179116	-6.17	0.000	.8373402	.9296567
_cons	915.6479	160.7499	38.85	0.000	582.5545	1439.198

Note: **_cons** estimates baseline odds.

. margins r.Gender, atmeans level(99)

Contrasts of adjusted predictions Number of obs = **1,509,190**
Model VCE: **Robust**

Expression: **Pr(y), predict()**
At: 0.Gender = **.8560175** (mean)
1.Gender = **.1439825** (mean)
1.nDecCases = **.0147225** (mean)
2.nDecCases = **.0360783** (mean)
3.nDecCases = **.0603131** (mean)
4.nDecCases = **.0668478** (mean)
5.nDecCases = **.0916836** (mean)
6.nDecCases = **.1077711** (mean)
7.nDecCases = **.1308477** (mean)
8.nDecCases = **.1488905** (mean)
9.nDecCases = **.1688237** (mean)

```
10.nDecCases = .1740218 (mean)
1.PSI        = .0292886 (mean)
2.PSI        = .0207727 (mean)
3.PSI        = .0029241 (mean)
4.PSI        = .1712415 (mean)
5.PSI        = .0112961 (mean)
6.PSI        = .0476666 (mean)
7.PSI        = .1273372 (mean)
8.PSI        = .0049344 (mean)
9.PSI        =  .109189 (mean)
10.PSI       = .2258967 (mean)
11.PSI       = .0586487 (mean)
12.PSI       = .0494782 (mean)
13.PSI       = .0370006 (mean)
14.PSI       = .0096741 (mean)
15.PSI       = .0717219 (mean)
16.PSI       = .0229295 (mean)
1.BinQtr     = .0833765 (mean)
2.BinQtr     = .0846182 (mean)
3.BinQtr     = .0776483 (mean)
4.BinQtr     = .0843916 (mean)
5.BinQtr     = .0846176 (mean)
6.BinQtr     = .0854021 (mean)
7.BinQtr     =   .08205 (mean)
8.BinQtr     = .0846719 (mean)
9.BinQtr     = .0835793 (mean)
10.BinQtr    = .0859521 (mean)
11.BinQtr    = .0821136 (mean)
12.BinQtr    = .0815789 (mean)
```

	df	chi2	P>chi2
Gender	1	3.89	0.0486

	Contrast	Delta-method std. err.	[99% conf. interval]	
Gender (Female vs Male)	-.0179467	.0091013	-.0413902	.0054967

```
. logistic y i.Gender##i.nDecCases i.PSI i.BinQtr [fweight = FreqWeights], vce(robust)      level(99)
```

```
Logistic regression                                     Number of obs = 1,509,190
                                                        Wald chi2(45) = 308385.47
                                                        Prob > chi2   =    0.0000
Log pseudolikelihood = -712513.8                        Pseudo R2     =    0.2549
```

y	Odds ratio	Robust std. err.	z	P>|z|	[99% conf. interval]	
Gender						
Female	1.1576	.2692683	0.63	0.529	.6358373	2.107516
nDecCases						
6 [5- 8]	.2043552	.0252386	-12.86	0.000	.1486709	.2808959
11 [9-13]	.0952839	.0114124	-19.63	0.000	.0699896	.1297196
16 [14-18]	.0574817	.0068445	-23.99	0.000	.0422987	.0781146
22 [19-25]	.0383681	.0045524	-27.48	0.000	.0282644	.0520836
30 [26-34]	.025859	.0030643	-30.84	0.000	.0190568	.0350892
41 [35-47]	.016094	.0019058	-34.87	0.000	.011863	.0218339
56 [48-66]	.0092468	.0010946	-39.56	0.000	.0068165	.0125435
82 [67-101]	.0048144	.0005699	-45.08	0.000	.0035491	.0065308
184 [>101]	.0021357	.0002529	-51.93	0.000	.0015743	.0028973

Gender#nDecCases						
Female# 6 [5- 8]	.9359747	.2263174	-0.27	0.784	.5020785	1.744844
Female#11 [9-13]	.8496172	.1999369	-0.69	0.489	.4634167	1.557668
Female#16 [14-18]	.8764163	.2054822	-0.56	0.574	.4791048	1.60321
Female#22 [19-25]	.7761227	.1813596	-1.08	0.278	.425135	1.416883
Female#30 [26-34]	.6968821	.1626611	-1.55	0.122	.3819864	1.271366
Female#41 [35-47]	.6871786	.1602343	-1.61	0.108	.3768962	1.252903
Female#56 [48-66]	.698638	.1628097	-1.54	0.124	.3833179	1.273343
Female#82 [67-101]	.7111783	.1657639	-1.46	0.144	.3901534	1.296348
Female#184 [>101]	1.025812	.239334	0.11	0.913	.5624311	1.870968
PSI						
Colorectal Surgery	.3912282	.009633	-38.11	0.000	.3671858	.4168448
Gastroenterology	.2887331	.0155008	-23.14	0.000	.2514434	.3315529
General Surgery	.3366293	.0070701	-51.84	0.000	.3189018	.3553423
Gynecological Oncology	.2480669	.0065948	-52.44	0.000	.2316485	.2656491
Neurosurgery	.3531553	.0079303	-46.35	0.000	.3333078	.3741847
OB/GYN	.3585096	.0079256	-46.40	0.000	.3386649	.3795171
OMFS	.3512323	.0164042	-22.40	0.000	.3114208	.3961334
Ophthalmology	.0799592	.001716	-117.71	0.000	.0756591	.0845037
Orthopedic Surgery	.1990651	.0041422	-77.57	0.000	.1886763	.2100259
Otolaryngology	.1812853	.0039744	-77.89	0.000	.1713317	.1918172
Plastic Surgery	.3667087	.0082777	-44.44	0.000	.3459949	.3886627
Podiatric Surgery	.1747502	.0042076	-72.45	0.000	.1642414	.1859313
Surgical Oncology	.3300266	.0093848	-38.98	0.000	.3067172	.3551074
Urology	.244235	.005327	-64.63	0.000	.2308919	.2583492
Vascular Surgery	.3610069	.0091974	-39.99	0.000	.3380766	.3854926
BinQtr						
2	1.013511	.010165	1.34	0.181	.9876625	1.040035
3	.9111873	.0094415	-8.98	0.000	.8871892	.9358345
4	.9558744	.0096239	-4.48	0.000	.9314035	.9809882
5	.9898352	.0099772	-1.01	0.311	.9644665	1.015871
6	.9914834	.0099433	-0.85	0.394	.966199	1.017429
7	.9227055	.0093583	-7.93	0.000	.8989122	.9471285
8	.9235837	.0092866	-7.91	0.000	.8999702	.9478168
9	.9367327	.0094471	-6.48	0.000	.9127119	.9613857
10	.9520286	.0094916	-4.93	0.000	.9278911	.976794
11	.8871153	.0089434	-11.88	0.000	.8643751	.9104537
12	.8822919	.0089062	-12.41	0.000	.8596467	.9055336
_cons	915.6479	109.989	56.77	0.000	671.9738	1247.684

Note: **_cons** estimates baseline odds.

```
.         margins r.Gender, atmeans level(99)
```

Contrasts of adjusted predictions   Number of obs = **1,509,190**
Model VCE: **Robust**

Expression: **Pr(y), predict()**

```
At: 0.Gender     = .8560175 (mean)
    1.Gender     = .1439825 (mean)
    1.nDecCases  = .0147225 (mean)
    2.nDecCases  = .0360783 (mean)
    3.nDecCases  = .0603131 (mean)
    4.nDecCases  = .0668478 (mean)
    5.nDecCases  = .0916836 (mean)
    6.nDecCases  = .1077711 (mean)
    7.nDecCases  = .1308477 (mean)
    8.nDecCases  = .1488905 (mean)
    9.nDecCases  = .1688237 (mean)
    10.nDecCases = .1740218 (mean)
    1.PSI        = .0292886 (mean)
    2.PSI        = .0207727 (mean)
    3.PSI        = .0029241 (mean)
    4.PSI        = .1712415 (mean)
```

```
    5.PSI        = .0112961 (mean)
    6.PSI        = .0476666 (mean)
    7.PSI        = .1273372 (mean)
    8.PSI        = .0049344 (mean)
    9.PSI        =  .109189 (mean)
    10.PSI       = .2258967 (mean)
    11.PSI       = .0586487 (mean)
    12.PSI       = .0494782 (mean)
    13.PSI       = .0370006 (mean)
    14.PSI       = .0096741 (mean)
    15.PSI       = .0717219 (mean)
    16.PSI       = .0229295 (mean)
    1.BinQtr     = .0833765 (mean)
    2.BinQtr     = .0846182 (mean)
    3.BinQtr     = .0776483 (mean)
    4.BinQtr     = .0843916 (mean)
    5.BinQtr     = .0846176 (mean)
    6.BinQtr     = .0854021 (mean)
    7.BinQtr     =   .08205 (mean)
    8.BinQtr     = .0846719 (mean)
    9.BinQtr     = .0835793 (mean)
    10.BinQtr    = .0859521 (mean)
    11.BinQtr    = .0821136 (mean)
    12.BinQtr    = .0815789 (mean)
```

	df	chi2	P>chi2
Gender	1	133.49	0.0000

	Contrast	Delta-method std. err.	[99% conf.	interval]
Gender (Female vs Male)	-.0179467	.0015533	-.0219479	-.0139456

```
. logistic y i.Gender  i.nDecCases i.PSI i.BinQtr [fweight = FreqWeights], vce(cluster FNI) level(99)

Logistic regression                                     Number of obs = 1,509,190
                                                        Wald chi2(36) =   9383.99
                                                        Prob > chi2   =    0.0000
Log pseudolikelihood = -712732.08                       Pseudo R2     =    0.2546

                                                 (Std. err. adjusted for 551 clusters in FNI)
```

y	Odds ratio	Robust std. err.	z	P>|z|	[99% conf.	interval]
Gender						
Female	.8781194	.0417145	-2.74	0.006	.7769838	.9924192
nDecCases						
6 [5- 8]	.2017026	.0216362	-14.92	0.000	.153008	.2658942
11 [9-13]	.0910522	.0100583	-21.69	0.000	.0685037	.1210228
16 [14-18]	.0549533	.0063212	-25.22	0.000	.0408615	.0739048
22 [19-25]	.0356152	.0041742	-28.45	0.000	.0263345	.0481667
30 [26-34]	.0236183	.0028099	-31.48	0.000	.0173843	.0320878
41 [35-47]	.0147056	.0017668	-35.12	0.000	.0107915	.0200395
56 [48-66]	.0084839	.0010244	-39.50	0.000	.0062162	.0115789
82 [67-101]	.00444	.0005463	-44.03	0.000	.003234	.0060957
184 [>101]	.0020203	.0002708	-46.30	0.000	.0014305	.0028532
PSI						
Colorectal Surgery	.3903614	.0574243	-6.39	0.000	.2672419	.5702027
Gastroenterology	.2856001	.1067279	-3.35	0.001	.1090725	.7478272

General Surgery	.3330369	.0363543	-10.07	0.000	.2514076	.4411702
Gynecological Oncology	.2433039	.0381638	-9.01	0.000	.1624346	.3644346
Neurosurgery	.3514533	.0426252	-8.62	0.000	.2571535	.4803335
OB/GYN	.3582086	.0396249	-9.28	0.000	.2693949	.4763022
OMFS	.3485518	.0886563	-4.14	0.000	.1810203	.6711312
Ophthalmology	.0799018	.0123512	-16.35	0.000	.0536579	.1189816
Orthopedic Surgery	.1979356	.0204248	-15.70	0.000	.1517363	.2582013
Otolaryngology	.1811975	.023318	-13.27	0.000	.1300747	.2524128
Plastic Surgery	.3627214	.0468783	-7.85	0.000	.2600136	.5059998
Podiatric Surgery	.1741491	.020742	-14.67	0.000	.1281394	.2366791
Surgical Oncology	.3237894	.0494655	-7.38	0.000	.2184556	.4799127
Urology	.2444228	.0246834	-13.95	0.000	.1884392	.3170386
Vascular Surgery	.358988	.0558366	-6.59	0.000	.2404825	.5358909
BinQtr						
2	1.014444	.0119061	1.22	0.222	.9842347	1.04558
3	.9118645	.0146763	-5.73	0.000	.8748339	.9504627
4	.956677	.0138945	-3.05	0.002	.9215482	.9931448
5	.9899713	.0146957	-0.68	0.497	.9528323	1.028558
6	.9913457	.0158125	-0.54	0.586	.9514409	1.032924
7	.9228297	.01599	-4.63	0.000	.8825477	.9649503
8	.9233178	.0163772	-4.50	0.000	.8820821	.9664813
9	.9365476	.0182405	-3.37	0.001	.8907222	.9847307
10	.9519871	.0174345	-2.69	0.007	.9081217	.9979714
11	.8873389	.0175948	-6.03	0.000	.8431556	.9338375
12	.8821533	.0179211	-6.17	0.000	.8371785	.9295442
_cons	993.9264	157.7549	43.48	0.000	660.3898	1495.919

Note: **_cons** estimates baseline odds.

```
.         margins r.Gender, atmeans level(99)
```

Contrasts of adjusted predictions   Number of obs = **1,509,190**
Model VCE: **Robust**

Expression: **Pr(y), predict()**

```
At: 0.Gender     = .8560175 (mean)
    1.Gender     = .1439825 (mean)
    1.nDecCases  = .0147225 (mean)
    2.nDecCases  = .0360783 (mean)
    3.nDecCases  = .0603131 (mean)
    4.nDecCases  = .0668478 (mean)
    5.nDecCases  = .0916836 (mean)
    6.nDecCases  = .1077711 (mean)
    7.nDecCases  = .1308477 (mean)
    8.nDecCases  = .1488905 (mean)
    9.nDecCases  = .1688237 (mean)
    10.nDecCases = .1740218 (mean)
    1.PSI        = .0292886 (mean)
    2.PSI        = .0207727 (mean)
    3.PSI        = .0029241 (mean)
    4.PSI        = .1712415 (mean)
    5.PSI        = .0112961 (mean)
    6.PSI        = .0476666 (mean)
    7.PSI        = .1273372 (mean)
    8.PSI        = .0049344 (mean)
    9.PSI        =  .109189 (mean)
    10.PSI       = .2258967 (mean)
    11.PSI       = .0586487 (mean)
    12.PSI       = .0494782 (mean)
    13.PSI       = .0370006 (mean)
    14.PSI       = .0096741 (mean)
    15.PSI       = .0717219 (mean)
    16.PSI       = .0229295 (mean)
    1.BinQtr     = .0833765 (mean)
    2.BinQtr     = .0846182 (mean)
    3.BinQtr     = .0776483 (mean)
```

```
    4.BinQtr     = .0843916 (mean)
    5.BinQtr     = .0846176 (mean)
    6.BinQtr     = .0854021 (mean)
    7.BinQtr     =   .08205 (mean)
    8.BinQtr     = .0846719 (mean)
    9.BinQtr     = .0835793 (mean)
    10.BinQtr    = .0859521 (mean)
    11.BinQtr    = .0821136 (mean)
    12.BinQtr    = .0815789 (mean)
```

	df	chi2	P>chi2
Gender	1	7.03	0.0080

	Contrast	Delta-method std. err.	[99% conf. interval]	
Gender (Female vs Male)	-.0253786	.0095684	-.050025	-.0007321

```
. logistic y i.Gender  i.nDecCases i.PSI          [fweight = FreqWeights], vce(cluster FNI) level(99)
```

```
Logistic regression                                 Number of obs = 1,509,190
                                                    Wald chi2(25) =   8621.11
                                                    Prob > chi2   =    0.0000
Log pseudolikelihood = -712966.13                   Pseudo R2     =    0.2544

                                        (Std. err. adjusted for 551 clusters in FNI)
```

y	Odds ratio	Robust std. err.	z	P>|z|	[99% conf. interval]	
Gender						
Female	.8767796	.0416362	-2.77	0.006	.7758317	.9908624
nDecCases						
6 [5- 8]	.2014596	.0215988	-14.94	0.000	.1528458	.2655353
11 [9-13]	.0909275	.0100473	-21.70	0.000	.0684044	.1208666
16 [14-18]	.0548673	.0063098	-25.24	0.000	.0408005	.0737837
22 [19-25]	.0355762	.004166	-28.49	0.000	.0263125	.0481013
30 [26-34]	.023608	.0028068	-31.51	0.000	.0173803	.0320673
41 [35-47]	.0147045	.001765	-35.15	0.000	.0107937	.0200322
56 [48-66]	.0084891	.0010241	-39.53	0.000	.0062217	.011583
82 [67-101]	.0044417	.0005459	-44.08	0.000	.0032364	.0060957
184 [>101]	.0020258	.000271	-46.36	0.000	.0014353	.0028592
PSI						
Colorectal Surgery	.3905234	.0575118	-6.38	0.000	.2672406	.5706788
Gastroenterology	.285621	.1058484	-3.38	0.001	.1099569	.7419213
General Surgery	.3331981	.0364451	-10.05	0.000	.2513872	.4416335
Gynecological Oncology	.243291	.0381702	-9.01	0.000	.1624115	.3644477
Neurosurgery	.3517324	.042692	-8.61	0.000	.2572957	.4808306
OB/GYN	.3587716	.0397615	-9.25	0.000	.2696743	.4773057
OMFS	.3491569	.0883845	-4.16	0.000	.1819049	.6701885
Ophthalmology	.0799673	.0123709	-16.33	0.000	.0536852	.1191161
Orthopedic Surgery	.1980468	.0204732	-15.66	0.000	.1517487	.2584703
Otolaryngology	.1813623	.0233896	-13.24	0.000	.1300998	.2528236
Plastic Surgery	.3627103	.0469347	-7.84	0.000	.2598988	.5061922
Podiatric Surgery	.1744503	.0207933	-14.65	0.000	.1283316	.2371427
Surgical Oncology	.3234106	.0493366	-7.40	0.000	.2183234	.4790801
Urology	.2447149	.0247705	-13.91	0.000	.1885501	.31761
Vascular Surgery	.359343	.0559021	-6.58	0.000	.2407025	.5364605
_cons	940.2858	149.3236	43.11	0.000	624.6085	1415.506

```
Note: _cons estimates baseline odds.

.         margins r.Gender, atmeans level(99)

Contrasts of adjusted predictions                    Number of obs = 1,509,190
Model VCE: Robust

Expression: Pr(y), predict()
At: 0.Gender     = .8560175 (mean)
    1.Gender     = .1439825 (mean)
    1.nDecCases  = .0147225 (mean)
    2.nDecCases  = .0360783 (mean)
    3.nDecCases  = .0603131 (mean)
    4.nDecCases  = .0668478 (mean)
    5.nDecCases  = .0916836 (mean)
    6.nDecCases  = .1077711 (mean)
    7.nDecCases  = .1308477 (mean)
    8.nDecCases  = .1488905 (mean)
    9.nDecCases  = .1688237 (mean)
    10.nDecCases = .1740218 (mean)
    1.PSI        = .0292886 (mean)
    2.PSI        = .0207727 (mean)
    3.PSI        = .0029241 (mean)
    4.PSI        = .1712415 (mean)
    5.PSI        = .0112961 (mean)
    6.PSI        = .0476666 (mean)
    7.PSI        = .1273372 (mean)
    8.PSI        = .0049344 (mean)
    9.PSI        =  .109189 (mean)
    10.PSI       = .2258967 (mean)
    11.PSI       = .0586487 (mean)
    12.PSI       = .0494782 (mean)
    13.PSI       = .0370006 (mean)
    14.PSI       = .0096741 (mean)
    15.PSI       = .0717219 (mean)
    16.PSI       = .0229295 (mean)
```

	df	chi2	P>chi2
Gender	1	7.20	0.0073

	Contrast	Delta-method std. err.	[99% conf. interval]	
Gender (Female vs Male)	-.0256869	.009572	-.0503428	-.001031

```
. logistic y i.Gender  i.nDecCases                [fweight = FreqWeights], vce(cluster FNI) level(99)

Logistic regression                                  Number of obs = 1,509,190
                                                     Wald chi2(10) =   6556.72
                                                     Prob > chi2   =    0.0000
Log pseudolikelihood = -740685.28                    Pseudo R2     =    0.2254
```

(Std. err. adjusted for **551** clusters in **FNI**)

y	Odds ratio	Robust std. err.	z	P>|z|	[99% conf. interval]	
Gender						
Female	**.8393106**	**.0324202**	**-4.54**	**0.000**	**.7598218**	**.9271151**
nDecCases						
6 [5- 8]	**.2072956**	**.0225362**	**-14.47**	**0.000**	**.1566655**	**.2742878**
11 [9-13]	**.0957119**	**.0107195**	**-20.95**	**0.000**	**.0717262**	**.1277185**
16 [14-18]	**.0576655**	**.0066968**	**-24.57**	**0.000**	**.0427565**	**.0777731**
22 [19-25]	**.036252**	**.0042721**	**-28.15**	**0.000**	**.0267609**	**.0491091**
30 [26-34]	**.0234019**	**.0027898**	**-31.50**	**0.000**	**.0172143**	**.0318136**
41 [35-47]	**.0142991**	**.0017186**	**-35.34**	**0.000**	**.0104919**	**.0194878**
56 [48-66]	**.0081872**	**.0009884**	**-39.80**	**0.000**	**.005999**	**.0111736**
82 [67-101]	**.0041366**	**.0005123**	**-44.31**	**0.000**	**.0030067**	**.005691**
184 [>101]	**.0014556**	**.0001936**	**-49.13**	**0.000**	**.0010335**	**.0020502**
_cons	**241.5786**	**30.31519**	**43.73**	**0.000**	**174.8554**	**333.7627**

Note: **_cons** estimates baseline odds.

```
.         margins r.Gender, atmeans level(99)
```

Contrasts of adjusted predictions   Number of obs = **1,509,190**
Model VCE: **Robust**

Expression: **Pr(y), predict()**
At: 0.Gender   = **.8560175** (mean)
    1.Gender   = **.1439825** (mean)
    1.nDecCases = **.0147225** (mean)
    2.nDecCases = **.0360783** (mean)
    3.nDecCases = **.0603131** (mean)
    4.nDecCases = **.0668478** (mean)
    5.nDecCases = **.0916836** (mean)
    6.nDecCases = **.1077711** (mean)
    7.nDecCases = **.1308477** (mean)
    8.nDecCases = **.1488905** (mean)
    9.nDecCases = **.1688237** (mean)
    10.nDecCases = **.1740218** (mean)

	df	chi2	P>chi2
Gender	**1**	**19.50**	**0.0000**

	Contrast	Delta-method std. err.	[99% conf. interval]	
Gender (Female vs Male)	**-.0349496**	**.0079151**	**-.0553375**	**-.0145617**

```
. logistic y i.Gender  i.nDecCases               [fweight = FreqWeights], vce(robust)      level(99)

Logistic regression                                     Number of obs = 1,509,190
                                                        Wald chi2(10) = 292441.60
                                                        Prob > chi2   =    0.0000
Log pseudolikelihood = -740685.28                       Pseudo R2     =    0.2254
```

y	Odds ratio	Robust std. err.	z	P>|z|	[99% conf.	interval]
Gender						
Female	.8393106	.0052974	-27.75	0.000	.8257757	.8530674
nDecCases						
6 [5- 8]	.2072956	.0219945	-14.83	0.000	.1577236	.2724478
11 [9-13]	.0957119	.0098627	-22.77	0.000	.0733994	.1248071
16 [14-18]	.0576655	.0059115	-27.83	0.000	.044283	.0750921
22 [19-25]	.036252	.003703	-32.48	0.000	.0278653	.0471628
30 [26-34]	.0234019	.0023872	-36.81	0.000	.0179944	.0304346
41 [35-47]	.0142991	.0014574	-41.68	0.000	.0109975	.0185919
56 [48-66]	.0081872	.0008341	-47.17	0.000	.0062975	.010644
82 [67-101]	.0041366	.0004214	-53.88	0.000	.0031819	.0053776
184 [>101]	.0014556	.0001483	-64.11	0.000	.0011196	.0018924
_cons	241.5786	24.59238	53.90	0.000	185.8572	314.0056

Note: **_cons** estimates baseline odds.

```
.         margins r.Gender, atmeans level(99)

Contrasts of adjusted predictions                       Number of obs = 1,509,190
Model VCE: Robust

Expression: Pr(y), predict()
At: 0.Gender     = .8560175 (mean)
    1.Gender     = .1439825 (mean)
    1.nDecCases  = .0147225 (mean)
    2.nDecCases  = .0360783 (mean)
    3.nDecCases  = .0603131 (mean)
    4.nDecCases  = .0668478 (mean)
    5.nDecCases  = .0916836 (mean)
    6.nDecCases  = .1077711 (mean)
    7.nDecCases  = .1308477 (mean)
    8.nDecCases  = .1488905 (mean)
    9.nDecCases  = .1688237 (mean)
    10.nDecCases = .1740218 (mean)
```

	df	chi2	P>chi2
Gender	1	731.16	0.0000

	Contrast	Delta-method std. err.	[99% conf.	interval]
Gender (Female vs Male)	-.0349496	.0012925	-.0382789	-.0316203

```
. logistic y i.Gender            i.PSI          [fweight = FreqWeights], vce(cluster FNI) level(99)

Logistic regression                                     Number of obs = 1,509,190
                                                        Wald chi2(16) =   1852.72
                                                        Prob > chi2   =    0.0000
Log pseudolikelihood = -838669.6                        Pseudo R2     =    0.1229

                                             (Std. err. adjusted for 551 clusters in FNI)
```

y	Odds ratio	Robust std. err.	z	P>|z|	[99% conf.	interval]
**Gender**						
Female	1.251476	.0633456	4.43	0.000	1.098498	1.425758
**PSI**						
Colorectal Surgery	.1645067	.0337072	-8.81	0.000	.0970443	.2788669
Gastroenterology	.4133091	.2238992	-1.63	0.103	.1023921	1.668337
General Surgery	.12735	.0212942	-12.32	0.000	.0827841	.1959076
Gynecological Oncology	.087914	.0188485	-11.34	0.000	.0506079	.1527205
Neurosurgery	.2014843	.0367827	-8.78	0.000	.1258979	.3224511
OB/GYN	.5610438	.1041451	-3.11	0.002	.3478093	.9050079
OMFS	.6050119	.1672364	-1.82	0.069	.2968543	1.233061
Ophthalmology	.0255215	.0050028	-18.71	0.000	.0154036	.0422854
Orthopedic Surgery	.0694477	.0116139	-15.95	0.000	.045142	.1068403
Otolaryngology	.1100405	.0219492	-11.06	0.000	.0658291	.1839447
Plastic Surgery	.2103785	.0407705	-8.04	0.000	.127705	.3465729
Podiatric Surgery	.331527	.0613568	-5.97	0.000	.2058179	.5340163
Surgical Oncology	.1608096	.0357806	-8.21	0.000	.0906578	.2852454
Urology	.2181288	.0375385	-8.85	0.000	.1400225	.3398038
Vascular Surgery	.3041456	.0652939	-5.54	0.000	.1749548	.5287337
_cons	16.39974	2.74388	16.72	0.000	10.65786	25.23505

Note: **_cons** estimates baseline odds.

```
.         margins r.Gender, atmeans level(99)

Contrasts of adjusted predictions                       Number of obs = 1,509,190
Model VCE: Robust

Expression: Pr(y), predict()
At: 0.Gender = .8560175 (mean)
    1.Gender = .1439825 (mean)
    1.PSI    = .0292886 (mean)
    2.PSI    = .0207727 (mean)
    3.PSI    = .0029241 (mean)
    4.PSI    = .1712415 (mean)
    5.PSI    = .0112961 (mean)
    6.PSI    = .0476666 (mean)
    7.PSI    = .1273372 (mean)
    8.PSI    = .0049344 (mean)
    9.PSI    =  .109189 (mean)
    10.PSI   = .2258967 (mean)
    11.PSI   = .0586487 (mean)
    12.PSI   = .0494782 (mean)
    13.PSI   = .0370006 (mean)
    14.PSI   = .0096741 (mean)
    15.PSI   = .0717219 (mean)
    16.PSI   = .0229295 (mean)
```

	df	chi2	P>chi2
Gender	1	21.23	0.0000

	Contrast	Delta-method std. err.	[99% conf. interval]	
Gender (Female vs Male)	**.0454518**	**.0098655**	**.0200398**	**.0708637**
